# Self-Guided Internet-Based Mindfulness-Informed Stress Management for Generalized Anxiety Disorder: Randomized Controlled Trial With Longitudinal Network Analysis

**DOI:** 10.2196/91751

**Published:** 2026-06-05

**Authors:** Ziwei Wang, Hui Qi Tong, Jianrong Yue, Siyan Wu, Ye Xia, Han Zhang, Yuan Yang

**Affiliations:** 1 Department of Neurology and Psychiatry, Tongji Hospital, Tongji Medical College, Huazhong University of Science and Technology, 1095 Jiefang Avenue, Wuhan, Hubei, 430030, China, 86 13995561816; 2 Hubei Key Laboratory of Neural Injury and Functional Reconstruction, Huazhong University of Science and Technology, Wuhan, Hubei, China; 3 Department of Psychiatry and Behavioral Sciences, Stanford University, Palo Alto, CA, United States

**Keywords:** generalized anxiety disorder, internet-based intervention, mindfulness, stress management, randomized controlled trial, longitudinal network analysis

## Abstract

**Background:**

Generalized anxiety disorder (GAD) is characterized by stress-anxiety reinforcement cycles. Evidence for brief, self-guided, internet-based stress management as an adjunct to pharmacotherapy remains limited.

**Objective:**

This study aimed to evaluate the efficacy of an 8-week self-guided, internet-based stress management program (iSM) incorporating mindfulness techniques as an adjunct to treatment as usual (TAU) in adults with GAD and to examine individual predictors of response and symptom dynamics.

**Methods:**

A single-blind, parallel-group, superiority randomized controlled trial was conducted at Tongji Hospital, Wuhan, China. A total of 140 adults with GAD were randomly assigned to iSM+TAU (n=73) or TAU (n=67). Outcome assessors were blinded to group allocation. The iSM intervention consisted of 8 weekly self-guided online modules integrating mindfulness-based training and Baduanjin-based stretching exercises. TAU comprised routine pharmacotherapy. Primary outcomes were posttreatment changes in anxiety and depressive symptoms. Secondary outcomes included sleep quality, somatic symptoms, social functioning, mindfulness, rumination, and perceived stress. Exploratory cross-lagged panel network (CLPN) and random-intercept cross-lagged panel modeling (RI-CLPM) analyses were used to examine temporal symptom dynamics.

**Results:**

All 140 randomized participants were included in the intention-to-treat analysis. Compared with TAU, iSM+TAU showed greater reductions at posttreatment in anxiety (Cohen *d*=−0.277, 95% CI −0.521 to −0.033) and depressive symptoms (Cohen *d*=−0.309, 95% CI −0.592 to −0.026). Significant between-group differences were also observed in somatic symptoms (Cohen *d*=−0.340, 95% CI −0.604 to −0.077), state anxiety (Cohen *d*=−0.537, 95% CI −0.849 to −0.224), mindfulness (Cohen *d*=0.666, 95% CI 0.327-1.006), rumination (Cohen *d*=−0.344, 95% CI −0.626 to −0.062), and perceived stress (Cohen *d*=−0.429, 95% CI −0.725 to −0.133), but not in sleep quality or social functioning. No serious adverse events were reported. Median session completion was 7 of 8. In exploratory analyses, higher baseline acting with awareness predicted greater treatment response (standardized β=0.167; 95% CI 0.031-0.335), whereas higher trait anxiety predicted poorer outcomes (standardized β=−0.150; 95% CI −0.234 to −0.002). CLPN and RI-CLPM identified 2 key within-person pathways, a bidirectional association between perceived stress (mean β=0.219; *P*<.001) and state anxiety (mean β=0.165; *P*=.02), and a unidirectional effect of mindfulness on subsequent anxiety reduction (mean β=–0.285; *P*<.001).

**Conclusions:**

To our knowledge, this is the first trial to evaluate a brief, self-guided, culturally adapted digital mindfulness intervention as an adjunct to pharmacotherapy in Chinese adults with GAD. The intervention showed clinically meaningful benefits and practical potential as a scalable, resource-efficient approach. Unlike prior studies focused mainly on symptom outcomes alone, this trial combined a randomized design with CLPN and RI-CLPM analyses to provide preliminary insight into symptom change processes over time, adding a more process-oriented analytic perspective that may inform future intervention refinement.

## Introduction

Generalized anxiety disorder (GAD) is a prevalent psychiatric condition affecting approximately 3.7% of the global population [1]. Beyond its hallmark symptoms of persistent worry and tension, GAD is increasingly conceptualized as involving central impairments in stress regulation mechanisms [2,3]. Patients with GAD exhibit heightened physiological and psychological stress responses, impaired stress recovery, and maladaptive stress appraisal patterns [3,4]. This chronic stress reactivity not only triggers anxiety episodes but also maintains the disorder through self-reinforcing stress-anxiety cycles [3,5]. Understanding GAD through this stress-centered lens suggests that interventions targeting stress management may address core maintaining mechanisms rather than merely alleviating downstream symptoms [6].

At the time of this writing, first-line treatments for GAD include pharmacotherapy and cognitive behavioral therapy (CBT) [7]. While pharmacological interventions provide symptomatic relief, they primarily target downstream manifestations rather than upstream stress processes, and adverse effects, including sedation and withdrawal, often limit long-term adherence [8,9]. Relapse rates of 56% following medication discontinuation further suggest that pharmacotherapy alone does not adequately address the disorder [10]. CBT, which includes cognitive restructuring as well as behavioral and physiological regulation techniques, is a well-established first-line treatment for GAD [7,11]. However, its uptake in routine care remains limited by constraints in training and supervision, underdeveloped service infrastructure, and practical barriers to patient participation [12,13]. Scalable adjunctive interventions that further strengthen stress management and coping capacity in daily life may therefore still have important clinical value. Stress management interventions incorporating mindfulness techniques have demonstrated promise for anxiety disorders [14]. Mindfulness-based stress reduction (MBSR), a well-established program that helps patients cope with chronic stress, teaches skills for recognizing and adaptively responding to stress rather than reacting automatically [15]. Meta-analyses support small-to-moderate effects on anxiety symptoms [14,16,17]. Mindfulness-based programs, including some web-based formats, also appear generally safe and well tolerated [18]. However, traditional face-to-face programs remain underused due to barriers including limited therapist availability, geographic constraints, and scheduling inflexibility [19]. Cultural factors further influence treatment engagement; for instance, Chinese individuals may be less accustomed to group-based psychological interventions involving open discussion of internal experiences. In resource-constrained settings like China, where mental health professionals number only 2.19 per 100,000 population, scalable stress management solutions are urgently needed [20,21].

Internet-delivered self-guided interventions offer a promising solution for expanding access to stress management training [22,23]. Digital formats eliminate therapist requirements, enabling immediate scalability without staffing constraints. Mindfulness-based interventions are theoretically well suited to target core maintaining mechanisms of GAD, including attentional dysregulation, worry reactivity, and experiential avoidance [16,24]. Core skills such as sustained attention and decentering from perseverative thinking may be strengthened through structured self-guided practice, as standardized sequential delivery can facilitate consolidation in daily life without continuous therapist support [15,25]. Several randomized controlled trials (RCTs) support efficacy for internet-based programs targeting individuals with GAD, although most evaluated stand-alone interventions [26-28]. Evidence supporting brief, self-guided, internet-based stress management as an adjunct to first-line pharmacotherapy for GAD remains limited. To address this gap, we developed an 8-week internet-delivered stress management program integrating mindfulness-based techniques with culturally adapted mind-body exercises (Baduanjin), designed specifically for self-guided use by Chinese adults with GAD.

Despite growing interest in digital stress management interventions, several critical gaps remain. First, most studies prioritize symptom reduction while overlooking broader functional outcomes such as sleep quality and social functioning [29], and evidence from non-Western clinical populations receiving pharmacological treatment remains scarce. Second, limited understanding of individual predictors constrains efforts toward personalized intervention [30]. Third, although theoretical models suggest that stress reduction mediates anxiety improvement, the temporal dynamics of stress-anxiety interactions during treatment remain underexplored [31]. Clarifying how these constructs influence each other over time—and how interventions disrupt maladaptive patterns—may uncover core mechanisms and guide optimization. Emerging longitudinal network approaches offer promising tools in this regard [32,33]. Specifically, cross-lagged panel network (CLPN) analysis can be used to examine directional associations among symptoms over time [34,35], whereas the random intercept cross-lagged panel model (RI-CLPM) more explicitly accounts for stable between-person differences and thereby provides a more stringent test of whether key associations are consistent with within-person temporal dynamics [36,37]. Together, these methods may help generate hypotheses about temporal pathways of symptom change and identify clinically relevant processes that may inform future intervention refinement and personalization.

In this study, we conducted an RCT evaluating an 8-week internet-based, self-guided stress management program as an adjunct to treatment as usual (TAU) among adults with GAD. We hypothesized that the intervention would reduce anxiety symptoms and that perceived stress and mindfulness would serve as key processes underlying symptom change. We also used regression analyses to identify predictors of treatment response and combined CLPN with RI-CLPM to explore temporal dynamics of the hypothesized stress-anxiety pathway.

## Methods

### Trial Design and Setting

This study was a single-blind, parallel-group, superiority RCT evaluating an 8-week online self-help mindfulness-based stress management program as an adjunct to pharmacological treatment for patients with GAD. Participants were randomized at the individual level. The trial was prospectively registered at the Chinese Clinical Trial Registry (registration number: ChiCTR2300078470) on December 8, 2023, prior to enrollment of the first participant on December 20, 2023. This trial is reported in accordance with the CONSORT (Consolidated Standards of Reporting Trials) 2025 statement and the CONSORT-EHEALTH (Consolidated Standards of Reporting Trials of Electronic and Mobile Health Applications and Online Telehealth) extension for web-based and mobile health interventions [38,39]. The completed CONSORT 2025 and CONSORT-EHEALTH checklists are provided as Checklist 1 and Checklist 2, respectively. This was a single-center trial conducted at Tongji Hospital, Tongji Medical College, Huazhong University of Science and Technology, Wuhan, China. Participants were consecutively recruited from the outpatient anxiety and depression clinics across 3 campuses of Tongji Hospital (Main Campus, Optical Valley Branch, and Sino-French New City Branch) between December 2023 and September 2024. Follow-up for the final participant, including outcome and adverse event assessment, was completed in December 2024. The full study protocol was not publicly posted or separately preregistered before trial completion, but it is available from the corresponding author upon reasonable request.

### Ethical Considerations

The study was approved by the Ethics Committee of Tongji Hospital, Tongji Medical College, Huazhong University of Science and Technology (approval number: TJ-IRB20230617) and was conducted in accordance with the Declaration of Helsinki and relevant ethical guidelines. In April 2024, an approved protocol amendment (TJ-IRB202404034) added electroencephalography and magnetic resonance imaging assessments for mechanistic exploration; these measures were not part of the original protocol and are not reported here. Written informed consent was obtained from all participants after they received a full explanation of the study purpose, procedures, and potential risks. Study data were deidentified prior to analysis and were accessible only to authorized research personnel. No financial compensation was provided for participation. The screenshot from the course interface shown in Figure S1 in Multimedia Appendix 1 was included with the written informed consent of the individual depicted. No other identifiable participant information is included in the manuscript or supplementary materials.

### Eligibility Criteria

Participants were screened for eligibility according to the following inclusion and exclusion criteria. Inclusion criteria were as follows: (1) a primary diagnosis of GAD according to the *DSM-5 (Diagnostic and Statistical Manual of Mental Disorders* [Fifth Edition]); (2) a Hamilton Anxiety Rating Scale (HAMA) score of ≥7; (3) age ≥18 years; (4) access to a smartphone or computer with a stable internet connection; and (5) sufficient cognitive and behavioral ability to follow the intervention procedures and complete study assessments. Exclusion criteria were as follows: (1) a diagnosis of schizophrenia, bipolar disorder, major depressive disorder with suicide risk, or substance use disorder (eg, alcohol or drug abuse) within the past 6 months; (2) receipt of formal psychotherapy (eg, CBT, psychodynamic therapy) or structured mindfulness training within the past 6 months; (3) severe visual, auditory, or cognitive impairments; (4) use of psychotropic medication (eg, antidepressants, antipsychotics, and benzodiazepines) within the past 3 months prior to enrollment; (5) pregnant, planned pregnancy, or breastfeeding; (6) concurrent participation in another clinical trial or enrollment of a first-degree relative in this study; (7) inability to cooperate in the intervention or data collection process; and (8) any other condition that, in the opinion of the investigators, would interfere with participation or data integrity. The primary diagnosis of GAD was established by the treating psychiatrist according to *DSM-5* criteria during routine psychiatric evaluation. Inclusion criteria were used for eligibility screening. Major psychiatric exclusion criteria were assessed during the same evaluation based on clinical interview, clinical history, and available medical records. Detailed procedures are provided in Multimedia Appendix 1.

### Procedures

At baseline, all participants underwent a comprehensive assessment, including demographic information, clinical history, and psychological evaluations. Eligible participants were randomly assigned to either the internet-based stress management program (iSM) plus TAU group or the TAU group. The iSM+TAU group received internet-based mindfulness stress management combined with routine pharmacological treatment, while the TAU group received only pharmacological treatment. Assessments were conducted at baseline (T0), midintervention (T1, Week 4), postintervention (T2, Week 8), and at 3-month follow-up (T3) to evaluate efficacy and safety, including psychological assessments, medication adjustments, adherence, and adverse event monitoring.

### Intervention

#### TAU Intervention

TAU was selected as the comparator to reflect real-world clinical practice and to evaluate the incremental benefit of the digital stress management program when added to standard pharmacological care. Participants in the TAU group received pharmacological treatment primarily based on selective serotonin reuptake inhibitors. Short-term benzodiazepines (≤4 wk) were permitted when clinically indicated, under physician supervision. Medication selection and dosage adjustments were made by attending psychiatrists in accordance with standard clinical practice, based on each patient’s clinical presentation, treatment needs, and tolerability. To support engagement and ensure comparable contact exposure across groups, the research team maintained participant communication through a designated WeChat (Tencent) group. Interactions with TAU participants were strictly limited to data collection and follow-up reminders, with no psychological support or behavioral guidance provided. To ensure ethical fairness, participants in the TAU group were offered access to the iSM after the study concluded. However, any data generated from this posttrial intervention were outside the scope of this analysis.

#### Mindfulness-Based Stress Management Program

Participants in the iSM+TAU group received an 8-week internet-delivered mindfulness-based stress management program in addition to the same pharmacological treatment provided to the TAU group. The intervention was delivered through a dedicated online learning platform, which provided structured weekly modules comprising instructional videos, audio-guided meditations, and daily mindfulness practice assignments. Participants were required to complete a 40‐60-minute core session each week, which served as a prerequisite for accessing the following session. The course emphasized progressive skill acquisition, covering core elements of mindfulness such as nonjudgmental present-moment awareness, breath-focused attention, and open monitoring. The weekly content followed a progressive thematic structure, and details of the weekly curriculum are provided in Table S1 of Multimedia Appendix 1. The first 4 sessions encompassed the core components of mindfulness training. Therefore, completion of at least 4 sessions was prespecified as the minimum threshold for adequate intervention exposure. All participants in the iSM group received the same prerecorded modules and standardized weekly content, while a separate WeChat group provided reminders and asynchronous technical support only, without individualized mindfulness guidance, live coaching, or psychotherapy. Individual progress was continuously monitored through the learning management system, which captured module completion status, video viewing duration, number of completed sessions, and cumulative practice time as indicators of intervention engagement (Figure S2 in Multimedia Appendix 1). Apart from the structured intervention modules, communication was limited to administrative and technical matters and kept consistent between study arms.

The mindfulness-based stress management program was adapted from the MBSR course originally developed by Kabat-Zinn [40] and was developed, reviewed, and adapted with input from a certified MBSR instructor. The prerecorded sessions were delivered by an experienced mindfulness therapist who had received standardized training in mindfulness-based practice. Compared to traditional face-to-face mindfulness programs, this intervention was shortened in duration to improve feasibility and adherence, making it more suitable for a self-paced, internet-based format. To enhance cultural relevance and accessibility, stretching exercises were adapted from Baduanjin (commonly known as the Eight Section Brocade), a traditional Chinese Qigong practice consisting of 8 gentle movements aimed at supporting flexibility, balance, and physical well-being instead of using conventional yoga-based stretching. All components of the intervention were delivered in a fully self-guided format without real-time therapist involvement. No structured psychotherapy, formal mindfulness training, or other concomitant interventions that could affect study outcomes were permitted during the trial.

#### Outcomes

Baseline demographic and clinical data included age, sex, BMI, education, residence type, marital status, childhood parental separation, employment, living situation, and medication use.

The primary outcomes were improvement in anxiety and depression from baseline to posttreatment (8 wk), measured with the Hamilton Anxiety Rating Scale [41] (HAMA; overall severity of anxiety symptoms) and the 21-item Hamilton Depression Rating Scale [42] (HAMD-21; overall severity of depressive symptoms), with additional assessments at midtreatment (4 wk) and 3-month follow-up to evaluate treatment trajectory and durability of effects.

Secondary outcomes were assessed at all time points, including state anxiety (State Anxiety Inventory [S-AI]) [43], sleep quality (Pittsburgh Sleep Quality Index [PSQI]) [44], mindfulness (Five Facet Mindfulness Questionnaire [FFMQ]) [45], somatic symptoms (the 15-item Patient Health Questionnaire [PHQ-15]) [46], social functioning (Social Disability Screening Schedule [SDSS]) [47], rumination (Ruminative Responses Scale [RRS]) [48], and perceived stress (the 10-item Perceived Stress Scale [PSS-10]) [49]. The public trial registration listed the primary outcomes and selected secondary outcomes but did not fully reflect the complete questionnaire-based longitudinal assessment battery used in the study. Several registered objective assessments were supplementary rather than core longitudinal outcomes and are not reported here because data completeness was insufficient for reliable analysis.

Several psychological trait measures were collected at baseline only, including trait anxiety (Trait Anxiety Inventory [T-AI]) [43], perceived security (Security Questionnaire [SQ]) [50], perceived social support (Perceived Social Support Scale [PSSS]) [51], loneliness (UCLA Loneliness Scale [UCLA-LS]) [52], interpersonal trust (Interpersonal Trust Scale [ITS]) [53], and social avoidance and distress (Social Avoidance and Distress Scale [SAD]) [54]. Detailed descriptions of each scale, scoring procedures, and psychometric properties are provided in the Multimedia Appendix 1.

#### Harms

Adverse events were assessed at each scheduled study assessment during the intervention and follow-up periods through participant self-report and routine clinical inquiry. Participants were asked about any unintended negative experiences during the study, including symptom worsening, physical discomfort, or other clinically relevant concerns. Medication selection and adjustment, including switching medications because of tolerability or clinical response, were allowed within TAU and were managed by the treating psychiatrists according to routine clinical practice. Reported adverse events were reviewed by the study psychiatrist, who assessed their severity and their likely relation to the internet-based intervention or concurrent pharmacotherapy on the basis of clinical judgment. Serious adverse events were defined as clinically significant deterioration requiring emergency psychiatric or medical evaluation or hospitalization, including suicidality or self-harm, severe worsening of anxiety or depressive symptoms, psychotic symptoms, or other acute conditions requiring immediate clinical intervention. Serious adverse events and withdrawals related to adverse events were also specifically recorded throughout the trial.

#### Randomization and Blinding

Participants were randomly assigned in a 1:1 ratio using simple randomization with a computer-generated sequence in R (R Core Team). The randomization sequence was generated and held by an independent researcher who was not involved in participant recruitment, outcome assessment, or intervention delivery. Eligible participants were enrolled by study investigators. The allocation sequence was not accessible to recruiting staff before assignment and was released only after eligibility had been confirmed and baseline assessment had been completed. Outcome assessors were not involved in intervention delivery and did not have access to the allocation sequence. The trial adopted a single-blind design; participants were necessarily aware of their allocation due to the nature of the intervention, but outcome assessors were blinded. To maintain blinding during analysis, datasets were anonymized and coded prior to statistical evaluation.

#### Patient and Public Involvement

Patients and members of the public were not involved in the design, conduct, reporting, or dissemination planning of this trial.

### Sample Size and Statistical Analysis

#### Sample Size Calculation

The sample size calculation was based on the primary outcome of anxiety symptom improvement. Using G*Power 3.1 (Heinrich Heine University, Düsseldorf) and informed by a previous study [55], we conservatively assumed a medium effect size (Cohen *d*=0.50). With a 2-tailed α=.05 and 80% power, a minimum of 128 participants (64 per group) was required. Allowing for a 20% dropout rate, the study was preregistered with a recruitment target of 160 participants. Due to funding and time constraints, 140 participants were ultimately randomized (iSM+TAU: n=73; TAU: n=67). This sample size exceeded the minimum required and provided approximately 84% power to detect an effect size of *d*=0.50 under unequal group sizes (2-tailed α=.05).

#### Statistical Analysis

All statistical analyses were conducted using SPSS (version 26.0; IBM Corp) and R (version 4.5.1; R Core Team). The primary analysis followed the intention-to-treat (ITT) principle, including all randomized participants analyzed in their assigned groups. Missing data patterns were assessed using Little's missing completely at random test [56]. Missing data were then handled using multiple imputation under the assumption that the data were missing at random. Per-protocol (PP) analyses were conducted as sensitivity analyses among participants who completed the study according to protocol. In the iSM+TAU group, the PP sample included participants who completed at least 4 intervention sessions and provided posttreatment primary outcome data. In the TAU group, the PP sample included participants who completed the posttreatment primary outcome assessment. No interim analyses were planned or conducted, and no stopping guidelines were prespecified. All tests were 2-sided with a significance level of α=.05, and effect estimates were reported with 95% CIs where appropriate. Adverse events were summarized descriptively.

#### Intervention Effects

Linear mixed effects modeling was used to account for the hierarchical structure of the data, with repeated measurements (time points) nested within individuals, using the lmerTest package developed by Kuznetsova, Brockhoff, and Christensen. The models included Group (iSM+TAU vs TAU), Time (T0, T1, T2, and T3), and the group×time interaction as fixed effects, with a random intercept for participants. Effect sizes were estimated using Cohen *d* derived from the estimated marginal means.

#### Predictors of Responses

Pearson or Spearman correlation analyses were performed to examine associations between baseline variables and primary outcomes. Variables showing significant associations (*P*<.05) were entered into multiple linear regression models, adjusting for demographic characteristics. These analyses were conducted on an exploratory basis.

#### Network and Temporal Dynamics Analysis

A 2-step exploratory analytic strategy was used to examine temporal symptom associations and to further evaluate selected pathways under a model that more explicitly accounts for stable between-person differences. All analyses were performed in R version 4.5.1 using the *bootnet*, *qgraph*, and *glmnet packages* for network estimation and the *lavaan* and *semPlot* packages for structural equation modeling.

First, CLPNs were estimated across 3 intervals (T0-T1, T1-T2, and T2-T3) using least absolute shrinkage and selection operator (LASSO) regression to explore directional associations among 6 core variables (HAMA, HAMD-21, FFMQ, PSQI, S-AI, and PSS-10). These variables were selected a priori to represent the hypothesized stress-management pathway and key GAD-related symptom domains. Confidence intervals for edge estimates were obtained using nonparametric bootstrap resampling. Network stability was evaluated using 3000 case-dropping bootstraps, and edges present in >90% of samples were considered stable.

Second, RI-CLPMs were constructed to further examine selected pathways identified in the CLPN analysis in a framework that more explicitly accounts for stable between-person differences through random intercepts. The models applied stationarity constraints and were estimated using robust maximum likelihood estimation. Model adequacy was evaluated using conventional fit indices, comparative fit index (CFI>0.90), root-mean-square error of approximation (RMSEA<0.08), and standardized root-mean-square residual (SRMR<0.08). Detailed model specifications and estimation procedures are provided in the Multimedia Appendix 1.

## Results

### Sample Characteristics

**Figure 1.**
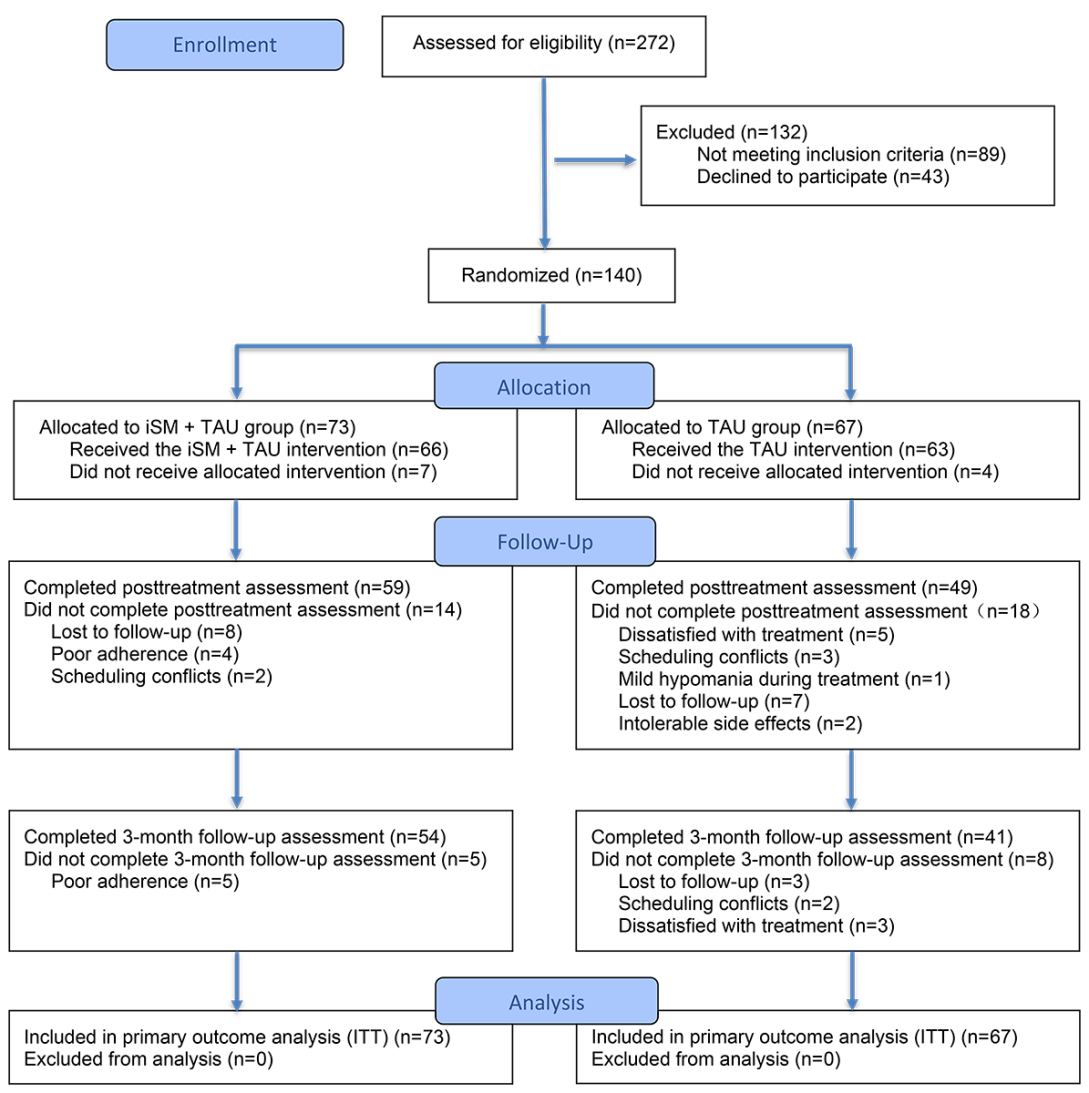
CONSORT (Consolidated Standards of Reporting Trials) flow diagram of participants with generalized anxiety disorder (GAD) in the randomized controlled trial conducted at Tongji Hospital. Participants were screened for eligibility, randomized to the internet-based stress management+treatment as usual (iSM+TAU) group or the TAU group, and followed through the intervention period, posttreatment assessment, 3-month follow-up, and inclusion in the final analyses, with reasons for not receiving the allocated intervention and loss to follow-up. GAD: generalized anxiety disorder; iSM: internet-based stress management program; ITT: intention-to-treat; TAU: treatment as usual.

Of 272 participants screened, 140 eligible participants were randomized to iSM+TAU (n=73) or TAU (n=67; Figure 1). Following randomization, 7 participants in the iSM+TAU group and 4 in the TAU group withdrew before initiating the intervention. At posttreatment, 59 participants in the iSM+TAU group and 49 in the TAU group completed the primary outcome assessment. By the 3-month follow-up, 54 participants in the iSM+TAU group and 41 in the TAU group had completed all assessments. Of the 73 participants randomized to the iSM+TAU group, 74% (54/73) completed at least 4 sessions, and the median number of sessions completed in the full intervention sample was 7 (IQR 3‐8). Missing data rates were 15.7% (22/140) at midintervention (4 wk, T1), 22.9% (32/140) at posttreatment (8 wk, T2), and 32.1% (45/140) at the 3-month follow-up (T3). Across all postbaseline assessments, 99 of 420 expected observations were missing, corresponding to an overall missingness rate of 23.6%. Little missing completely at random test was nonsignificant (*χ²*_172_=184.2; *P*=.25), indicating no significant departure from missing at random.

No significant differences were observed in any baseline characteristics between the iSM+TAU and TAU groups (all *P*>.05; Table S2 in Multimedia Appendix 1). The median age of all participants was 33 (IQR 26‐39) years, and 66.4% (93/140) were female. The sample was predominantly urban and relatively well educated, 81.4% (114/140) were from urban areas, and 77.9% (109/140) had a college education or above. Medication use was similar between the 2 groups (*P*>.05), with selective serotonin reuptake inhibitor monotherapy being the most common regimen, accounting for 74% (54/73) in the iSM+TAU group and 70.1% (47/67) in the TAU group.

### Primary Outcome Analysis

**Figure 2.**
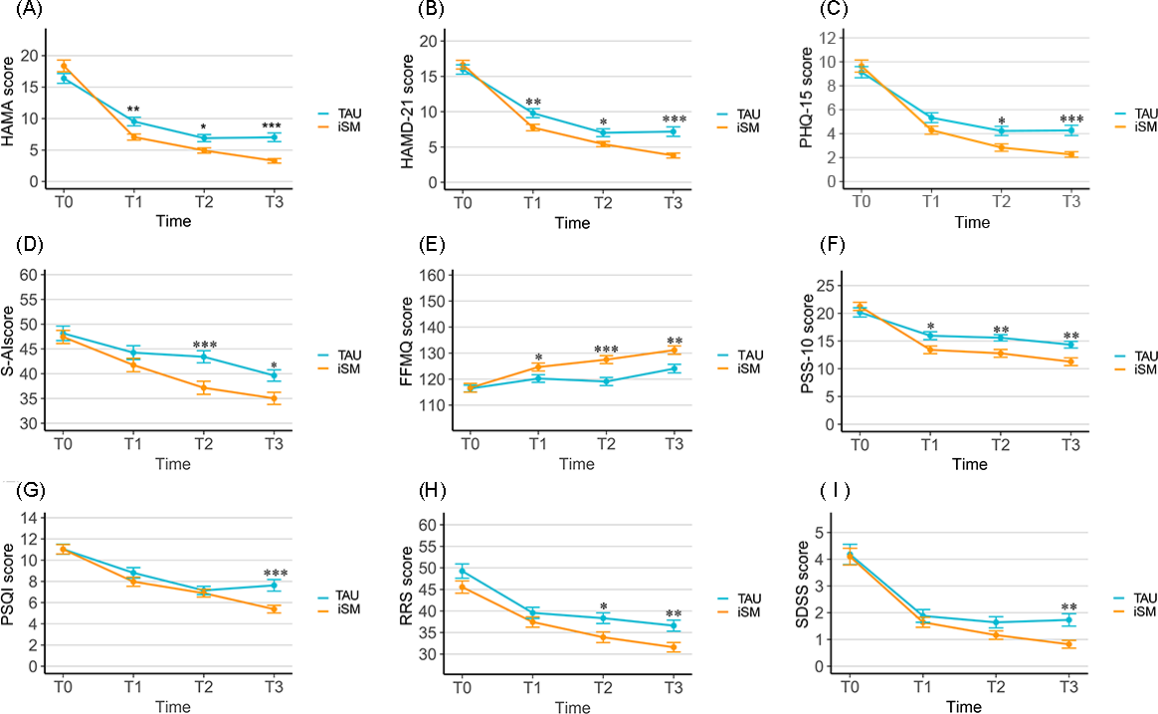
Changes in primary and secondary outcome measures over time among participants with generalized anxiety disorder (GAD) in the intention-to-treat (ITT) analysis of the randomized controlled trial at Tongji Hospital (N=140). Panels show longitudinal changes in (A) anxiety, (B) depression, (C) somatic symptoms, (D) state anxiety, (E) mindfulness, (F) perceived stress, (G) sleep quality, (H) rumination, and (I) functional impairment from baseline (T0) to midintervention (T1), posttreatment (T2), and 3-month follow-up (T3). Error bars represent standard errors of the mean. FFMQ: Five Facet Mindfulness Questionnaire; HAMA: Hamilton Anxiety Rating Scale; HAMD-21: 21-item Hamilton Depression Rating Scale; ITT: intention-to-treat; iSM: internet-based stress management program; PHQ-15: 15-item Patient Health Questionnaire; PSQI: Pittsburgh Sleep Quality Index; PSS-10: 10-item Perceived Stress Scale; RRS: Ruminative Responses Scale; SDSS: Social Disability Screening Schedule; S-AI: State Anxiety Inventory; TAU: treatment as usual; *: *P*<.05; **: *P*<.01; ***: *P*<.001.

All randomized participants (n=140) were included in the primary ITT analyses. Linear mixed-effects models showed significant group×time interactions for both anxiety and depressive symptoms (*P*<.001). At posttreatment (T2), the iSM+TAU group demonstrated significantly greater reductions in anxiety symptoms (HAMA: mean difference [MD]=−1.994, 95% CI −3.758 to −0.230; *P*=.03; Cohen *d*=−0.277, 95% CI −0.521 to −0.033) and depressive symptoms (HAMD-21: MD=−1.649, 95% CI −3.165 to −0.133; *P*=.03; Cohen *d*=−0.309, 95% CI −0.592 to −0.026) compared with the TAU group. These between-group differences further increased at the 3-month follow-up, reaching moderate effect sizes for both HAMA (Cohen *d*=−0.511, 95% CI −0.755 to −0.267) and HAMD-21 (Cohen *d*=−0.637, 95% CI −0.920 to −0.355), as illustrated in Figures 2A and 2B. Detailed estimates are provided in Table S3 in Multimedia Appendix 1, and consistent findings were observed in the PP sensitivity analysis (Table S4 in Multimedia Appendix 1).

### Secondary Outcome Analysis

In the ITT sample, significant group×time interactions were found for PHQ-15, PSQI, S-AI, FFMQ, and PSS-10 (Table S3 in Multimedia Appendix 1). At posttreatment, the iSM+TAU group showed significant improvements in somatic symptoms (PHQ-15: MD=−1.388, 95% CI −2.465 to −0.312; *P*=.01; Cohen *d*=−0.340, 95% CI −0.604 to −0.077; Figure 2C), state anxiety (S-AI: MD=−6.254, 95% CI −9.905 to −2.602; *P*<.001; Cohen *d*=−0.537, 95% CI −0.849 to −0.224; Figure 2D), mindfulness level (FFMQ: MD=8.433, 95% CI 4.119-12.748; *P*<.001; Cohen *d*=0.666, 95% CI 0.327-1.006; Figure 2E), and perceived stress (PSS-10: MD=−2.801, 95% CI −4.740 to −0.863; *P*=.005; Cohen *d*=−0.429, 95% CI −0.725 to −0.133; Figure 2F). Sleep quality (PSQI: MD=−0.259, 95% CI −1.479 to 0.962; *P*=.68; Cohen *d*=−0.068, 95% CI −0.386 to 0.251) showed no significant between-group difference at posttreatment but demonstrated a significant effect at follow-up (MD=−2.243, 95% CI −3.464 to −1.023, *P*<.001; Cohen *d*=−0.588, 95% CI −0.906 to −0.269; Figure 2G).

Rumination (RRS: MD=−4.424, 95% CI −8.069 to −0.780; *P*=.02; Cohen *d*=−0.344, 95% CI −0.626 to −0.062) showed significant posttreatment reductions, although the overall group×time interaction was not significant (*P*=.38; Figure 2H). Social functioning measured by the SDSS showed no significant between-group difference at posttreatment (MD=−0.424, 95% CI −1.091 to 0.243; *P*=.21; Cohen *d*=−0.146, 95% CI −0.376 to 0.083), with significant effects emerging only at follow-up (MD=−0.947, 95% CI −1.614 to −0.279; *P*=.006; Cohen *d*=−0.327, 95% CI −0.557 to −0.097; Figure 2I). The PP sensitivity analysis revealed consistent patterns of change across all secondary outcomes (Table S4 in Multimedia Appendix 1).

### Harms

No serious adverse events were reported during the trial. A limited number of participants (15/140, 10.7%) reported mild transient dizziness or nausea, which was considered more likely related to concurrent pharmacotherapy than to the internet-based behavioral intervention. No adverse events led to study withdrawal.

### Dose-Response Relationship and Treatment Adherence

Of the 73 participants randomized to the iSM+TAU group, 7 (9.6%) did not complete a full session, 5 (6.8%) completed 1 session, 4 (5.5%) completed 2 sessions, 3 (4.1%) completed 3 sessions, 8 (11%) completed 4 sessions, 5 (6.8%) completed 5 sessions, 4 (5.5%) completed 6 sessions, 11 (15.1%) completed 7 sessions, and 26 (35.6%) completed all 8 sessions (Table S5 in Multimedia Appendix 1). The mean number of sessions completed in the full intervention sample was 5.3 (SD 2.9), and the median number of sessions completed was 7 (IQR 3‐8).

We then conducted exploratory analyses to examine whether greater intervention exposure was associated with better clinical outcomes. Among participants who completed all follow-up assessments, those who completed ≥4 sessions showed significantly greater anxiety reduction than those completing <4 sessions (HAMA: *F*_1,47_=5.13; *P*=.03), but no significant difference was observed for depressive symptoms (HAMD-21: *F*_1,47_=2.86; *P*=.10; Table 1). In addition, compared with the TAU group, participants in the iSM+TAU subgroup who did not complete the intervention showed no significant differences in either anxiety symptoms (HAMA: *F*_1,62_=0.31; *P*=.58) or depressive symptoms (HAMD-21: *F*_1,62_=0.01; *P*=.91; Table 1), suggesting that limited intervention exposure may have attenuated the potential treatment benefit.

**Table 1.** Comparison of primary outcomes according to intervention completion status among participants with generalized anxiety disorder (GAD) who completed the 3-month follow-up assessment (T3) at Tongji Hospital (n=95). Observed posttreatment scores are presented as mean (SD). Adjusted scores are presented as adjusted mean (SE), estimated using analysis of covariance for each pairwise comparison, controlling for age, sex, BMI, education level, marital status, and employment status.

Outcome measure	Completed versus noncompleted		Noncompleted versus TAU^a^	
	Completed (n=26)	Noncompleted (n=28)	Noncompleted (n=28)	TAU (n=41)
HAMA^b^				
Observed mean (SD)	3.46 (2.52)	5.61 (3.61)	5.61 (3.61)	6.05 (3.81)
Adjusted mean (SE)	3.56 (0.60)	5.51 (0.58)	5.53 (0.75)	6.10 (0.61)
*F* test (*df*)	5.13 (1, 47)	—^c^	0.31 (1, 62)	—
*P* value	.03	—	.58	—
HAMD-21^d^				
Observed mean (SD)	4.12 (2.93)	6.00 (3.39)	6.00 (3.39)	6.41 (4.49)
Adjusted mean (SE)	4.38 (0.56)	5.75 (0.54)	6.18 (0.81)	6.29 (0.66)
*F* test (*df*)	2.86 (1, 47)	—	0.01 (1, 62)	—
*P* value	.10	—	.91	—

a TAU: treatment as usual.

b HAMA: Hamilton Anxiety Rating Scale.

c Not applicable.

d HAMD-21: 21-item Hamilton Depression Rating Scale.

### Predictors of Treatment Response in the iSM+TAU Group

Among iSM completers (n=54), several adjusted regression models were used to examine baseline predictors of anxiety reduction (HAMA change from baseline to posttreatment). After univariate screening (*P*<.05) and adjustment for covariates including baseline HAMA scores, age, sex, marital status, education level, residential status, employment status, and childhood parental separation, separate covariate-adjusted linear regression models showed that higher trait anxiety was significantly associated with smaller reductions in anxiety symptoms (standardized β=–0.150, 95% CI –0.234 to –0.002; *P*=.046), while greater acting with awareness predicted larger improvements (standardized β=0.167, 95% CI 0.031-0.335; *P*=.02; Table 2). These findings suggest that individuals with lower trait anxiety and higher baseline mindful awareness may be more suitable for self-guided digital mindfulness interventions.

**Table 2.** Multiple linear regression analysis of baseline psychological traits predicting Hamilton Anxiety Rating Scale (HAMA) reduction among participants with generalized anxiety disorder (GAD) in the internet-based stress management+treatment as usual (iSM+TAU) group who completed at least 4 intervention sessions at Tongji Hospital (n=54). Models were adjusted for age, sex, baseline HAMA score, marital status, educational attainment, residential setting, employment status, and childhood parental separation.

Predictors	Standardized β coefficient (95% CI)	*P* value	Δ*R*²^a^	Model *R*²^b^
Trait anxiety (T-AI)^c^	–0.150 (–0.234 to –0.002)	.046	0.013	0.872
Acting with awareness (FFMQ)^d^	0.167 (0.031 to 0.335)	.02	0.017	0.876
Symptom rumination (RRS)^e^	–0.099 (–0.281 to 0.075)	.25	0.004	0.863
Perceived helplessness (PSS-10)^f^	–0.040 (−0.316 to 0.189)	.61	0.001	0.860
Lack of self-efficacy (PSS-10)	–0.121 (–0.708 to 0.022)	.07	0.011	0.870
Certainty control (SQ)^g^	0.044 (–0.143 to 0.254)	.57	0.001	0.860

a ΔR² indicates the incremental variance explained by each predictor.

b Model *R*² represents the total variance explained by each separately fitted adjusted model.

c T-AI: Trait Anxiety Inventory.

d FFMQ: Five Facet Mindfulness Questionnaire.

e RRS: Ruminative Responses Scale.

f PSS-10: 10-item Perceived Stress Scale.

g SQ: Security Questionnaire.

### CLPN Analysis

**Figure 3.**
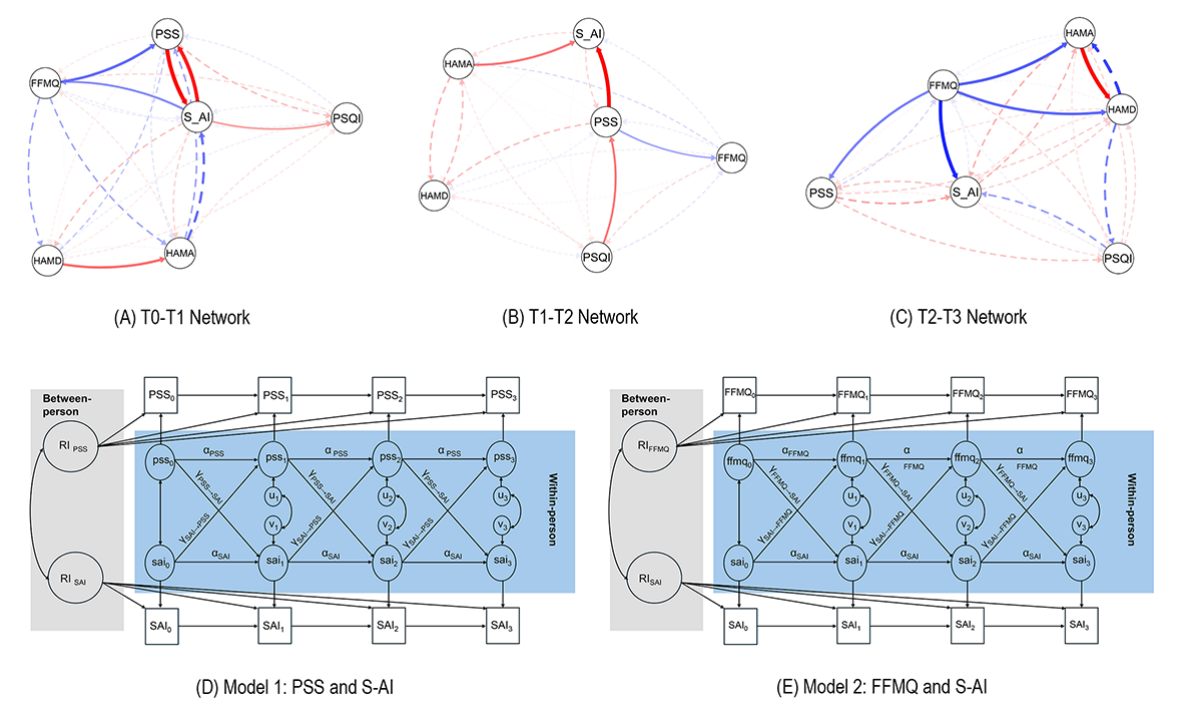
Temporal network dynamics and random-intercept cross-lagged panel models of psychological variables across 4 assessment time points in participants with generalized anxiety disorder (GAD) in this randomized controlled trial at Tongji Hospital. Panels show temporal cross-lagged panel networks from (A) T0 to T1, (B) T1 to T2, and (C) T2 to T3, and constrained random-intercept cross-lagged panel models for (D) Model 1, examining perceived stress and state anxiety, and (E) Model 2, examining mindfulness and state anxiety. FFMQ: Five Facet Mindfulness Questionnaire; GAD: generalized anxiety disorder; HAMA: Hamilton Anxiety Rating Scale; HAMD: Hamilton Depression Rating Scale; PSQI: Pittsburgh Sleep Quality Index; PSS: Perceived Stress Scale; S-AI: State Anxiety Inventory; T0: baseline; T1: midintervention; T2: postintervention; T3: 3-month follow-up.

Across the 3 intervals (T0-T1, T1-T2, and T2-T3), the temporal network structure revealed distinct dynamic patterns among the 6 retained variables: HAMA, HAMD-21, FFMQ, PSQI, S-AI, and PSS (Figure 3). In these temporal networks, nodes represent psychological variables, and directed edges indicate temporal predictive associations from time *t* to time *t*+1. Red edges denote positive associations, whereas blue edges denote negative associations. Solid lines indicate edges with at least 90% bootstrap support, whereas dashed lines indicate edges with less than 90% bootstrap support.

During the early treatment phase (T0-T1; Figure 3A), the strongest cross-lagged associations were observed between perceived stress and state anxiety, with PSS predicting subsequent S-AI (B=0.389, 95% CI 0.066-0.672) and S-AI, in turn, predicting PSS (B=0.351, 95% CI 0.084-0.570), suggesting a reciprocal temporal association between perceived stress and state anxiety. Higher baseline mindfulness (FFMQ) predicted lower subsequent perceived stress (B=−0.226, 95% CI −0.419 to −0.017), whereas higher state anxiety predicted reductions in mindfulness (B=−0.182, 95% CI −0.352 to −0.015). In addition, depressive symptoms predicted higher subsequent anxiety (HAMD → HAMA: B=0.237, 95% CI 0.058-0.501), and elevated state anxiety was associated with poorer subsequent sleep quality (S-AI → PSQI: B=0.164, 95% CI 0.093-0.323). In this interval, PSS showed the highest in-predictability, whereas S-AI exhibited the highest out-predictability (Figure S3 in Multimedia Appendix 1).

During midtreatment (T1-T2; Figure 3B), the PSS → S-AI pathway remained the strongest cross-lagged edge and further strengthened (B=0.442, 95% CI 0.214-0.621). Anxiety symptoms (HAMA) also showed a positive predictive effect on subsequent state anxiety (B=0.227, 95% CI 0.017-0.472). In parallel, poorer sleep quality predicted higher perceived stress (PSQI → PSS: B=0.205, 95% CI 0.003-0.408), while perceived stress predicted lower mindfulness (PSS → FFMQ: B=−0.151, 95% CI −0.322 to −0.044). State anxiety demonstrated the highest in- and out-predictability during this phase (Figure S3 in Multimedia Appendix 1).

During the consolidation phase (T2-T3; Figure 3C), the strongest cross-lagged association emerged between anxiety and depressive symptoms, with HAMA predicting higher subsequent HAMD-21 scores (B=0.311, 95% CI 0.086-0.481). Notably, mindfulness was negatively associated with subsequent state anxiety (B=−0.270, 95% CI −0.397 to −0.088), anxiety (B=−0.202, 95% CI −0.398 to −0.004), depression (B=−0.202, 95% CI −0.341 to −0.018), and perceived stress (B=−0.150, 95% CI −0.352 to −0.018). In this interval, depressive symptoms (HAMD-21) exhibited the highest in-predictability and out-predictability, suggesting that depressive symptoms were more strongly connected with subsequent symptom changes during the late phase (Figure S3 in Multimedia Appendix 1). Together, these findings suggest that higher mindfulness was associated with lower subsequent symptoms across multiple domains. Over time, the stress-anxiety pattern appeared to weaken, whereas the anxiety-depression linkage became more pronounced in the late phase.

The correlation stability coefficients for edge weights were 0.284 for T0-T1, 0.358 for T1-T2, and 0.358 for T2-T3, all exceeding the recommended minimum threshold of 0.25, indicating modest but acceptable stability of edge estimates across intervals. In contrast, correlation stability coefficients for in-strength and out-strength centrality were below 0.25 in most intervals, and these centrality metrics were therefore not interpreted.

### RI-CLPM Analyses

To reduce the risk of spurious causal inferences arising from stable between-person differences in conventional cross-lagged models, RI-CLPM analyses were conducted to isolate within-person lagged associations. Random intercepts capture stable between-person differences (trait-like components) across time. Squares and uppercase letters indicate observed variables, whereas ovals and lowercase letters denote latent within-person components. Autoregressive (α) paths represent temporal stability within constructs, and cross-lagged (γ) paths represent directional within-person effects between constructs across adjacent time points. Residual terms (u_₁_-u_₃_ and v_₁_-v_₃_) represent time-specific unexplained within-person variance. All autoregressive and cross-lagged paths are constrained to be equal across time points, estimating lagged effects from time *t* to time *t*+1 across 4 measurement waves. Only 2 pathways showed significant within-person effects: the bidirectional pathway between PSS and S-AI (Figure 3D) and the unidirectional pathway from FFMQ to S-AI (Figure 3E). Both models demonstrated good fit with appropriate variance partitioning between stable traits and temporal fluctuations (see Multimedia Appendix 1).

### Model 1: Perceived Stress and State Anxiety

Cross-lagged paths revealed robust bidirectional within-person associations (Figure 3D): greater perceived stress predicted higher subsequent state anxiety (b=0.363, 95% CI 0.163-0.563, *P*<.001; mean β=0.219), and higher state anxiety predicted greater subsequent stress (b=0.062, 95% CI 0.012-0.111, *P*=.02; mean β=0.165). Both effects exceeded the large effect size threshold (β≥0.12), supporting a reciprocal reinforcement mechanism at the individual level (Table 3).

**Table 3.** Regression parameter estimates from random-intercept cross-lagged panel models of perceived stress, mindfulness, and state anxiety among participants with generalized anxiety disorder (GAD) at Tongji Hospital.

Model and regression parameters	b^a^ (95% CI)	SE	Standardized β (range)	*P* value
Model 1:^b^ PSS^c^ and S-AI^d^				
Autoregressive effects				
PSS → PSS at t+1	0.018 (–0.098 to 0.133)	0.059	0.017 to 0.031	.77
S-AI → S-AI at t+1	–0.129 (–0.272 to 0.013)	0.073	–0.160 to –0.142	.08
Cross-lagged effects				
PSS → S-AI at *t*+1	0.363 (0.163 to 0.563)	0.102	0.164 to 0.288	<.001
S-AI → PSS at *t*+1	0.062 (0.012 to 0.111)	0.025	0.148 to 0.194	.02
Model 2:^b^ FFMQ and S-AI				
Autoregressive effects				
FFMQ^e^ → FFMQ at t+1	0.362 (0.193 to 0.531)	0.086	0.498 to 0.567	<.001
S-AI → S-AI at t+1	–0.124 (–0.266 to 0.019)	0.073	–0.160 to –0.129	.09
Cross-lagged effects				
FFMQ → S-AI at t+1	–0.210 (–0.326 to –0.093)	0.059	–0.312 to –0.262	<.001
S-AI → FFMQ at t+1	–0.067 (–0.164 to 0.031)	0.050	–0.108 to –0.064	.18

a b: unstandardized regression coefficient (constrained across time); β range, range of standardized coefficients across time points.

b Model 1 examined perceived stress and state anxiety. Model 2 examined mindfulness and state anxiety. Both models included random intercepts to account for between-person differences.

c PSS: Perceived Stress Scale.

d S-AI: State Anxiety Inventory.

e FFMQ: Five Facet Mindfulness Questionnaire.

### Model 2: Mindfulness and State Anxiety

A significant unidirectional within-person effect was observed (Figure 3E): higher mindfulness predicted lower subsequent state anxiety (b=−0.210, 95% CI −0.326 to −0.093; *P*<.001; mean β=−0.285), whereas state anxiety did not significantly predict later mindfulness (b=−0.067, 95% CI −0.164 to 0.031; *P*=.18). These findings suggest that increases in mindfulness precede reductions in state anxiety over time (Table 3).

## Discussion

### Principal Findings

This study evaluated a brief, self-guided, internet-delivered mindfulness-informed stress management program as an adjunct to TAU for patients with GAD. Overall, the intervention was associated with improvement in anxiety, depression, and several functional outcomes, while also showing acceptable adherence and feasibility in a real-world clinical setting. In addition, the longitudinal network-based analyses provided exploratory evidence suggesting that stress- and mindfulness-related symptom processes may be involved in how improvement unfolded over time.

The present study demonstrated significant anxiety reduction in the iSM+TAU group compared with TAU, with effects that were sustained and larger at the 3-month follow-up. This pattern of sustained and growing improvement suggests that skills acquired during training continue to consolidate after formal intervention ends [57]. Although the effect size was lower than that found in a comparable RCT with more intensive interventions in a therapist-guided online mindfulness intervention [55], it was larger than the small effects reported in a meta-analysis of online mindfulness-based interventions [22] and consistent with recent work suggesting that fully self-guided digital mindfulness approaches can be acceptable and beneficial for adults with GAD [58].

Beyond symptom reduction in anxiety and depression, iSM demonstrated broad functional improvements, including sleep quality, somatic symptoms, and social functioning. These multidimensional benefits are particularly important in real-world clinical settings where functional recovery often lags behind symptom improvement [59-62]. More broadly, mindfulness-based interventions may have effects across multiple domains rather than on emotional symptoms alone, with meta-analytic evidence suggesting benefits for global cognition and several cognitive control–related processes [63]. Although these domains were not directly examined in the present study, they may provide a broader framework for understanding symptom improvement. Notably, perceived stress, a central focus of the intervention, was significantly reduced by midtreatment, suggesting that stress reduction may be an early and clinically relevant component of improvement.

The retention rate of 77% (108/140) and median completion of 7 sessions demonstrate excellent feasibility for a self-directed intervention. Participant retention in the present trial was comparable to that reported by Li et al [64] and notably higher than rates documented in other large-scale RCTs of online mindfulness-based interventions, where attrition has reached approximately 38.7% [65]. Importantly, most dropouts occurred before or during the initial sessions, suggesting that early engagement is critical for retention [66]. The relatively high adherence to this self-guided format underscores the feasibility of iSM, particularly in settings with limited therapeutic resources [20,21]. Treatment adherence was closely associated with outcomes; participants who completed more sessions showed greater anxiety reduction, whereas those with incomplete participation derived little benefit beyond TAU. Dose-response analyses further indicated that completing at least 4 sessions, covering core mindfulness components, was associated with greater improvement and may represent a pragmatic minimum exposure target in self-guided settings [67].

Individual psychological characteristics appeared to significantly influence the effectiveness of the intervention. Higher baseline acting with awareness—a mindfulness facet reflecting the tendency to be attentive to present-moment activities—predicted greater improvements, consistent with previous research suggesting that existing mindfulness capacity facilitates stress management skill acquisition [68]. This may reflect better ability to engage with training exercises or greater openness to experiential approaches. Conversely, elevated trait anxiety was associated with attenuated benefits, potentially reflecting impaired attentional control that limits effective engagement with stress management techniques [69]. Although these predictor effects were modest, they may still be informative as partial contributors to treatment response variability. From a clinical perspective, these findings suggest that individuals with higher baseline mindfulness capacity may be well-suited for self-guided digital programs as adjuncts to standard care, whereas those with very elevated trait anxiety may require more intensive or therapist-supported interventions. Such stratification could maximize both clinical outcomes and health care efficiency.

While traditional RCT analyses established the overall efficacy of the intervention, network analyses were used to explore how symptom associations changed over time and to generate hypotheses about possible temporal processes underlying change. CLPN analyses suggested stage-dependent changes in the pattern of temporal symptom associations across the study period. In the initial phase, perceived stress and anxiety showed a relatively prominent bidirectional association, a pattern broadly consistent with network theories emphasizing persistent interconnections among symptoms despite reductions in overall symptom levels [70]. This pattern appeared to weaken over time, whereas anxiety remained highly connected and stress and sleep continued to show temporal associations with other variables. In later phases, depressive symptoms appeared more strongly embedded in the temporal network and were more often linked to subsequent changes in other variables. Meanwhile, higher mindfulness was generally associated with lower subsequent levels of stress, anxiety, and depression across phases. These findings are broadly consistent with nonlinear models of psychological change, which propose that symptom improvement may occur through stage-dependent reorganization [71].

However, not all temporal associations identified in the CLPN analyses remained evident under the RI-CLPM framework. RI-CLPM, which more explicitly accounts for stable trait-like between-person differences, suggested that 2 pathways remained relatively robust under this more stringent model and were broadly compatible with within-person temporal dynamics. First, the stress-anxiety relationship was bidirectional: perceived stress predicted subsequent anxiety, and anxiety predicted subsequent stress, a pattern broadly consistent with reciprocal processes described in transactional stress models [72]. Second, mindfulness showed a unidirectional protective effect on anxiety, whereas the reverse path was not supported in the model, suggesting that mindfulness may function as a protective regulatory process, although this interpretation remains inferential and model-based, consistent with attentional control theory [73]. Other associations identified in the CLPN analyses were not retained in the RI-CLPM framework, suggesting that they may be less robust as within-person temporal pathways. Taken together, these findings are broadly consistent with theoretical models emphasizing stress reactivity, attentional regulation, and stage-dependent symptom change and suggest that mindfulness-related processes may contribute to symptom improvement over time [31,72,73].

The present study provides new evidence for a brief, self-guided, culturally adapted internet-based mindfulness-informed stress management program evaluated as a feasible and clinically useful adjunct to pharmacological treatment for GAD in a Chinese clinical setting. Unlike prior work focused mainly on symptom outcomes alone, it also incorporated exploratory longitudinal analyses to provide a more process-oriented perspective on how symptoms may change over time, suggesting that perceived stress and mindfulness may represent potential processes involved in treatment response and warrant further investigation. The iSM may also help expand access to mental health care in settings with limited psychotherapy resources. As a brief digital program requiring only basic internet access and no therapist scheduling, iSM may offer a practical and relatively low-cost option for wider implementation, especially within stepped-care models and among underserved populations [74,75]. The integration of culturally relevant elements further enhances engagement and acceptability, supporting iSM’s potential as an equitable adjunct to traditional services within evolving mental health systems.

### Limitations

This study has several limitations. First, although participants were recruited across 3 hospital campuses, this study was conducted within a single hospital system with a relatively homogeneous sample, limiting generalizability. The sample profile may partly reflect self-selection into a self-guided digital intervention, which may be more readily adopted by younger and more educated participants. These factors highlight the need for future adaptations and testing in broader populations. In addition, the achieved sample size was slightly below the pre-registered recruitment target due to funding and time constraints. Future studies should expand the sample size and include multiple centers with more diverse populations across different regions, while also considering adaptations that may improve accessibility for broader clinical groups and enhance external validity. Additionally, the CLPN and RI-CLPM analyses were exploratory and should be interpreted cautiously. Future studies with larger samples are needed to verify the robustness and reproducibility of these longitudinal findings. Second, the follow-up period was limited to 3 months after the intervention, making it difficult to evaluate long-term effectiveness and adherence. Future research should extend the observation period and incorporate strategies such as booster sessions and individualized guidance to support sustained engagement and outcomes. Third, this study primarily relied on standardized scales for assessment, which may be subject to expectancy effects. Future research should incorporate objective physiological and neuroimaging measures to improve the precision of mechanism evaluation. Fourth, the network analysis was restricted to 6 theory-driven variables capturing the hypothesized stress-management pathway and key GAD-related symptom domains, which helped preserve model interpretability and stability given the sample size and repeated-measures design, but other potentially relevant mechanisms (eg, emotion regulation and self-efficacy) were not examined. Future studies with larger samples and broader assessments should investigate more comprehensive mechanistic networks. Fifth, the use of TAU rather than an active control also limits the ability to distinguish intervention-specific effects from nonspecific influences such as attention, expectancy, or engagement with a digital platform, although this design was consistent with the pragmatic aim of evaluating added benefit beyond usual care. Finally, while RI-CLPM strengthens within-person temporal inference by controlling for stable individual differences, causal conclusions still require experimental manipulation of specific mechanisms.

### Conclusion

To our knowledge, this is the first RCT to evaluate a brief, self-guided, culturally adapted digital mindfulness intervention as an adjunct to pharmacotherapy in Chinese adults with GAD. The intervention yielded clinically meaningful benefits across anxiety, depression, and several secondary outcomes related to stress, mindfulness, and associated clinical domains. Its fully self-guided format, culturally adapted content, and minimal infrastructure requirements highlight its practical potential as a scalable, resource-efficient approach in real-world settings where psychotherapy availability is limited. Unlike prior studies focused mainly on symptom outcomes alone, this trial combined a randomized design with exploratory CLPN and RI-CLPM analyses to provide preliminary insight into symptom change processes over time. The longitudinal findings suggested temporally distinct dynamics, with stress-anxiety coupling more prominent early in treatment and mindfulness-related pathways becoming more apparent in later phases. These findings contribute not only preliminary evidence of adjunctive clinical utility but also add a more process-oriented analytic perspective that may inform future intervention refinement.

## Supplementary material

10.2196/91751 Multimedia Appendix 1 This multimedia appendix provides detailed supplementary methods, including a comprehensive description of the internet-based mindfulness stress management intervention, full descriptions of all psychological measurement instruments and scoring procedures, and detailed analytic methods for the cross-lagged panel network and random-intercept cross-lagged panel modeling analyses. Supplementary figures and tables supporting the main results are also included.

10.2196/91751 Checklist 1 CONSORT 2025 reporting checklist.

10.2196/91751 Checklist 2 CONSORT-EHEALTH reporting checklist.

## Abbreviations

CBT: cognitive behavioral therapy
CFI: comparative fit index
CLPN: cross-lagged panel network
CONSORT: Consolidated Standards of Reporting Trials
CONSORT-EHEALTH: Consolidated Standards of Reporting Trials of Electronic and Mobile Health Applications and Online Telehealth
*DSM-5*: *Diagnostic and Statistical Manual of Mental Disorders* (Fifth Edition)
FFMQ: Five Facet Mindfulness Questionnaire
GAD: generalized anxiety disorder
HAMA: Hamilton Anxiety Rating Scale
HAMD-21: 21-item Hamilton Depression Rating Scale
iSM: internet-based stress management program
ITS: Interpersonal Trust Scale
ITT: intention-to-treat
LASSO: least absolute shrinkage and selection operator
MBSR: mindfulness-based stress reduction
MD: mean difference
PHQ-15: 15-item Patient Health Questionnaire
PP: per-protocol
PSQI: Pittsburgh Sleep Quality Index
PSS-10: 10-item Perceived Stress Scale
PSSS: Perceived Social Support Scale
RCT: randomized controlled trial
RI-CLPM: random-intercept cross-lagged panel model
RMSEA: root-mean-square error of approximation
RRS: Ruminative Responses Scale
S-AI: State Anxiety Inventory
SAD: Social Avoidance and Distress Scale
SDSS: Social Disability Screening Schedule
SQ: Security Questionnaire
SRMR: standardized root-mean-square residual
T-AI: Trait Anxiety Inventory
TAU: treatment as usual
UCLA-LS: UCLA Loneliness Scale
