# Supplementary material for: Self-Guided Internet-Based Mindfulness-Informed Stress Management for Generalized Anxiety Disorder: Randomized Controlled Trial With Longitudinal Network Analysis
Source: J Med Internet Res. 2026 Jun 5;28:e91751. doi: 10.2196/91751 (PMC13240990; doi:10.2196/91751)
Supplement: Multimedia Appendix 1 [file jmir-v28-e91751-s001.docx]

**Appendix**

**Supplementary Methods**

**Diagnostic and Eligibility Assessment Procedures**

Diagnostic Assessment

The primary diagnosis of generalized anxiety disorder (GAD) was established by the treating psychiatrist through routine psychiatric diagnostic and differential diagnostic evaluation, incorporating DSM-5 diagnostic criteria, structured clinical interview, psychiatric history, and review of available medical records. As the diagnosis was based on comprehensive clinician-administered assessment rather than a single screening instrument, sensitivity and specificity values associated with a standalone cutoff score are not directly applicable to the diagnostic procedure used in this trial. The Hamilton Anxiety Rating Scale (HAMA) threshold of ≥7 was applied solely as a study eligibility criterion to ensure that enrolled participants presented with at least mild anxiety symptom severity at baseline, and did not serve as the basis for diagnostic assignment.

Ascertainment of Exclusion Criteria

Exclusion criteria were ascertained through two complementary procedures. Major psychiatric exclusion conditions, including schizophrenia, bipolar disorder, major depressive disorder with suicide risk, and substance use disorder, were evaluated as part of the baseline psychiatric diagnostic and differential diagnostic process conducted by the treating psychiatrist, based on DSM-5 criteria, clinical interview, psychiatric history, and review of available medical records. Prior receipt of formal psychotherapy or structured mindfulness training within the past six months was determined through participant self-report at screening. Severe visual, auditory, or cognitive impairments were operationally defined as any impairment substantially interfering with independent use of internet-based materials or completion of self-report assessments, and were determined through clinician assessment during the baseline evaluation. Use of psychotropic medication within the past three months was verified through participant self-report and cross-checked against available prescription records where accessible. Current or planned pregnancy or breastfeeding was determined through participant self-report. Concurrent participation in another clinical trial, or enrollment of a first-degree relative in this study, was determined through participant self-report at screening. Inability to cooperate in the intervention or data collection process, and any other condition judged likely to interfere with participation or data integrity, were determined based on clinician judgment during the baseline assessment.

**Internet-Based Mindfulness Stress Management (iSM) Intervention**

Structure and Content of the iSM Program

The internet-based mindfulness stress management (iSM) program was adapted from the Mindfulness-Based Stress Reduction (MBSR) framework and was developed and facilitated by a certified MBSR instructor. Compared with standard MBSR programs, the intervention was shortened in duration to improve feasibility and adherence within a fully self-guided, online delivery format.

Given that traditional MBSR programs require substantial time commitments, prolonged training durations may reduce sustained engagement in internet-based self-help interventions. Therefore, while preserving the core therapeutic elements of mindfulness training, the overall duration and structure of the program were optimized to enhance accessibility and adherence.

To further improve cultural relevance, mindful movement practices were adapted by replacing traditional Western yoga-based stretching with Baduanjin, a form of traditional Chinese qigong. In the adapted iSM program, Baduanjin was incorporated as the mindful movement component rather than as a separate exercise module. During the first 4 weekly sessions, participants learned 2 movements per week through slow, step-by-step instruction. Throughout this process, they were guided to attend to breathing, bodily sensations, posture, and moment-to-moment experience, thereby integrating movement practice with core mindfulness principles. During the remaining 4 weeks, participants practiced the complete 8-movement sequence as an integrated mindful movement routine. This staged design was intended to reduce learning burden, improve adherence in a self-guided online format, and increase acceptability across different age groups and genders within the local cultural context. Further details of this component are provided in the subsection “Baduanjin-Based Mindful Movement Component” below.

The program adopted a progressive, theme-based structure, with each week focusing on a specific core topic (Table S1). The eight weekly modules were organized as follows: (1) cognitive defusion and awareness of automatic pilot (Week 1); (2) cognitive biases and emotional responses in anxiety (Week 2); (3) physiological mechanisms of anxiety as a stress response (Week 3); (4) emotion regulation using the R.A.I.N. technique (Week 4); (5) interpersonal anxiety and mindful communication (Week 5); (6) anxiety-related behavioral patterns and activity regulation (Week 6); (7) mechanisms of anxiety maintenance and relapse prevention (Week 7); and (8) integration of mindfulness into daily life and long-term practice (Week 8). Through this structured progression, participants were guided to gradually develop mindfulness skills and apply them to everyday experiences, with the goal of promoting sustained psychological well-being beyond the intervention period.

Participants assigned to the iSM group received an 8-week internet-based mindfulness stress management program in addition to treatment as usual (TAU), which consisted of stable pharmacotherapy identical to that received by the TAU-only group. The intervention was delivered through a dedicated online learning platform (https://ism-study.com.cn/), which provided structured access to mindfulness-based video lectures, guided audio practices, and home practice assignments.

The program followed a weekly modular structure, with one new session released each week. Participants were instructed to complete one session per week, with each session lasting approximately 40–60 minutes. In addition, participants were asked to engage in daily mindfulness practice on the remaining six days of each week, with a recommended practice duration of approximately 45 minutes per day. An overview of the user-facing course interface is shown in Figure S1.

To enhance adherence and engagement, a WeChat-based support group was established and moderated by the research team. Within this group, participants were able to ask questions related to course content and practice experiences. Instructors provided timely responses, shared practice reminders, and contacted participants via WeChat messages or phone calls when necessary to encourage completion of sessions and home practice.

The learning management system incorporated an automated backend that allowed researchers to monitor participants’ engagement in real time, including course progress, video viewing duration, and completion of practice assignments. These data enabled the research team to identify participants with low adherence and provide additional reminders or support as needed. An example of the backend monitoring interface is shown in Figure S2.

The main functions of the learning platform included: (1) a course homepage that displayed weekly learning objectives and sequentially unlocked course content (Figure S1); (2) integrated video and audio resources delivering mindfulness instruction and guided practices; (3) a learning feedback system that automatically recorded course progress, session completion, and practice duration; and (4) interactive support delivered via the WeChat group, where participants received guidance and encouragement throughout the intervention period.

Based on the structure of the iSM program, the first four weeks focused on the systematic training of core mindfulness skills, including the identification of automatic thought patterns, recognition of cognitive biases, awareness of bodily sensations, and emotion regulation using the R.A.I.N. technique (Recognize, Allow, Investigate, and Non-identification). These foundational components constitute the core of mindfulness-based interventions and are essential for developing basic awareness and self-regulation capacities. Accordingly, completion of at least the first four weekly sessions was defined as having received an adequate dose of the intervention for per-protocol analyses.

Baduanjin-Based Mindful Movement Component

Baduanjin is a traditional Chinese qigong exercise that has been practiced for centuries and remains widely recognized in Chinese culture as a gentle mind-body practice. It consists of 8 sequential movements, each combining coordinated breathing, postural regulation, and focused attention. In traditional teaching, the 8 movements are summarized in short classical phrases, with each posture emphasizing a different bodily region or functional focus.

The 8 Baduanjin movements included in the program were as follows:

1. Holding Up the Sky with Both Hands to Regulate the Sanjiao

This opening movement primarily engages the shoulders, arms, and upper back. It helps expand the chest, facilitates smoother breathing, and promotes flexibility in the upper body.

Chinese: 两手托天理三焦

Chinese pronunciation: Liǎng shǒu tuō tiān lǐ sān jiāo

2. Drawing the Bow to Shoot the Hawk

This movement emphasizes opening the chest and stretching the spine and waist. It may help improve trunk flexibility, postural control, and upper-body coordination.

Chinese: 左右开弓似射雕

Chinese pronunciation: Zuǒ yòu kāi gōng sì shè diāo

3. Lifting One Arm to Regulate the Spleen and Stomach

This posture stretches the lateral trunk and promotes spinal mobility. In traditional practice, it is considered beneficial for abdominal regulation and digestive function.

Chinese: 调理脾胃臂单举

Chinese pronunciation: Tiáo lǐ pí wèi bì dān jǔ

4. Looking Backwards to Relieve the Five Fatigues and Seven Injuries

This movement involves gentle turning of the head and upper body, which may reduce tension in the neck and shoulder region and improve cervical flexibility.

Chinese: 五劳七伤向后瞧

Chinese pronunciation: Wǔ láo qī shāng xiàng hòu qiáo

5. Punching with a Fierce Gaze to Increase Strength

This movement combines upper-limb extension with a focused gaze and stable posture. It may strengthen the arms and shoulders while enhancing concentration and bodily activation.

Chinese: 攒拳怒目增气力

Chinese pronunciation: Cuán quán nù mù zēng qì lì

6. Holding the Feet with Both Hands to Strengthen the Kidneys and Waist

This forward-bending movement stretches the lower back and posterior leg muscles, helping improve flexibility and relieve lumbar stiffness. In traditional terms, it is also considered to support vitality.

Chinese: 两手攀足固肾腰

Chinese pronunciation: Liǎng shǒu pān zú gù shèn yāo

7. Shaking the Head and Wagging the Tail to Clear Internal Heat

This posture involves coordinated movement of the trunk and pelvis with gentle shifting of body weight. It may help release tension in the lower back and promote whole-body relaxation.

Chinese: 摇头摆尾去心火

Chinese pronunciation: Yáo tóu bǎi wěi qù xīn huǒ

8. Rising on the Toes to Help Dispel Illness

This final movement involves gentle heel lifting or rising onto the toes. It is intended to stimulate circulation, activate the lower limbs, and conclude the sequence with a sense of bodily integration.

Chinese: 背后七颠诸病消

Chinese pronunciation: Bèi hòu qī diān zhū bìng xiāo

Collectively, these movements involve gentle stretching, trunk rotation, coordinated upper- and lower-limb movement, breathing regulation, and sustained attention to bodily sensations. In the present intervention, their function was not only physical exercise, but also the cultivation of mindful awareness through movement.

Key Adaptations From Standard Face-to-Face MBSR

The present intervention was adapted from standard face-to-face Mindfulness-Based Stress Reduction (MBSR) into a self-guided, internet-delivered stress management program for Chinese adults with GAD. While the program retained the 8-week structure and core mindfulness progression, several modifications were made to enhance feasibility, scalability, and cultural relevance.

First, therapist-led, real-time group delivery was replaced with standardized prerecorded digital modules, allowing consistent delivery while reducing reliance on therapist time.

Second, intensive in-person components of standard MBSR, such as full-day meditation practice, were not included. Group discussion and instructor-led inquiry were also omitted to maintain a fully self-guided format.

Third, the program was streamlined for online use, with weekly core sessions of approximately 40–60 minutes supported by audio-guided home practice and daily assignments, in order to improve accessibility and adherence in a self-paced setting.

Fourth, the movement component was culturally adapted by replacing conventional yoga-based stretching with Baduanjin, a traditional Chinese mind-body exercise more familiar to Chinese participants.

Despite these modifications, the program retained the core therapeutic elements of mindfulness training, including present-moment awareness, nonjudgmental observation of experience, breath-focused attention, and open monitoring.

**Detailed descriptions of measurement instruments**

**Primary outcome measures**

**Hamilton Anxiety Rating Scale (HAMA)**
Anxiety severity was assessed by trained clinicians using the Hamilton Anxiety Rating Scale. The HAMA consists of 14 items, each rated on a 5-point scale ranging from 0 (not present) to 4 (severe). Total scores range from 0 to 56, with higher scores indicating greater anxiety severity. According to Zhang’s psychiatric rating scale manual, the HAMA has been widely used in the assessment of anxiety symptoms in China and is considered to have good consistency and practical clinical utility[1].

**21-item Hamilton Depression Rating Scale (HAMD**-**21)**
Depressive symptoms were evaluated using the 21-item version of the Hamilton Depression Rating Scale. Items are rated on either a 0–4 or 0–2 scale, depending on symptom characteristics. Total scores range from 0 to 52, with higher scores reflecting more severe depressive symptoms. The Chinese version of the Hamilton Depression Rating Scale has shown acceptable psychometric properties, with satisfactory internal consistency (Cronbach α=0.71). Principal-component analysis identified a 5-factor structure accounting for 52.4% of the total variance, supporting acceptable construct validity[2].

**Secondary outcome measures**

**State Anxiety Inventory (S-AI)**
State anxiety was measured using the State Anxiety Inventory, a subscale of the State-Trait Anxiety Inventory (STAI). The S-AI comprises 20 items rated on a 4-point Likert scale (1–4), with higher scores indicating greater current anxiety. The Chinese Mandarin version has demonstrated excellent internal consistency (Cronbach α=0.91), with validity supported by a 4-factor structure and a significant correlation with the Chinese Hamilton Anxiety Rating Scale (r=0.69)[3].

**Five Facet Mindfulness Questionnaire (FFMQ)**
Mindfulness was assessed using the 39-item Five Facet Mindfulness Questionnaire. Items are rated on a 5-point Likert scale ranging from 1 to 5, with higher scores representing greater levels of mindfulness across its five facets. The Chinese version of the FFMQ has demonstrated acceptable overall psychometric performance in Chinese samples, with acceptable internal consistency overall[4]. Confirmatory factor analysis supported the original 5-factor structure, indicating acceptable construct validity (comparative fit index (CFI)=0.926, Tucker-Lewis index (TLI)=0.903, and root mean square error of approximation (RMSEA)=0.067)[4].

**Ruminative Responses Scale (RRS)**
Rumination was evaluated using the 22-item Ruminative Responses Scale. Each item is rated on a 4-point scale (1–4), with higher scores indicating a greater tendency toward ruminative thinking. The Chinese version of the RRS has demonstrated good psychometric properties in Chinese undergraduate samples, with excellent internal consistency for the total scale (Cronbach α=0.90) and good test-retest reliability (0.82)[5]. Confirmatory factor analysis supported an acceptable model fit, indicating acceptable construct validity (root mean square error of approximation (RMSEA)=0.068 and comparative fit index (CFI)=0.83)[5].

**10-item Perceived Stress Scale (PSS**-**10)**
Perceived stress was measured using the 10-item Perceived Stress Scale, which assesses the extent to which individuals perceive life situations as stressful. Items are rated on a 5-point scale from 0 to 4, with higher scores indicating greater perceived stress. The Simplified Chinese version of the PSS-10 has shown good internal consistency (Cronbach α=0.85) and acceptable construct validity, with a 2-factor structure accounting for 62.4% of the variance[6].

**Social Disability Screening Schedule (SDSS)**
Social functioning was assessed using the Social Disability Screening Schedule, consisting of 10 items rated on a 3-point scale (0–2). Higher scores indicate greater levels of social and functional impairment. According to data reported by Zhang et al., the SDSS showed good interrater reliability, with agreement rates of 85%-90% and kappa coefficients ranging from 0.6 to 1.0[1]. In the same report, SDSS total scores were correlated with Present State Examination scores (r=0.72–0.83), supporting acceptable criterion validity[1].

**15-item Patient Health Questionnaire (PHQ**-**15)**
Somatic symptom severity was evaluated using the Patient Health Questionnaire–15. Items are rated on a 3-point scale (0–2), with higher total scores reflecting more severe somatic complaints. The Chinese version of the PHQ-15 has shown good internal consistency (Cronbach α=0.83), with construct validity supported by a 3-factor structure accounting for 56% of the total variance[7].

**Pittsburgh Sleep Quality Index (PSQI)**
Sleep quality was assessed using the Pittsburgh Sleep Quality Index, which includes 19 items across seven components. Each component is scored on a 0–3 scale, and the global PSQI score ranges from 0 to 21. A total score greater than 5 indicates poor sleep quality. The Chinese version has demonstrated good internal consistency (Cronbach α=0.84) and acceptable criterion validity, with a strong correlation with the Insomnia Severity Index (r=0.842)[8].

**Additional psychological assessments (predictors and covariates)**

**Trait Anxiety Inventory (T-AI)**
Trait anxiety was measured using the Trait Anxiety Inventory, a subscale of the State-Trait Anxiety Inventory. The T-AI consists of 20 items rated on a 4-point scale (1–4), with higher scores indicating greater anxiety proneness. The Chinese Mandarin version has demonstrated excellent internal consistency for the trait anxiety subscale (Cronbach α=0.92), with validity supported by a 4-factor structure and a significant correlation with the Chinese Hamilton Anxiety Rating Scale (r=0.74)[3].

**Security Questionnaire (SQ)**
Perceived security was assessed using the Security Questionnaire, which comprises 16 items rated on a 5-point Likert scale (1–5). Higher scores reflect stronger perceived interpersonal security and sense of control. The Chinese SQ has demonstrated acceptable internal consistency (Cronbach α=0.80) and structural validity, with factor analysis supporting a 2-factor structure accounting for 46.4% of the variance[9].

**Perceived Social Support Scale (PSSS)**
Perceived social support was measured using the 12-item Perceived Social Support Scale. Items are rated on a 7-point scale (1–7), with higher scores indicating greater perceived social support. The Chinese version of the PSSS has demonstrated good internal consistency (Cronbach α=0.84), and construct validity was supported by a 3-dimensional structure accounting for 67.1% of the total variance[10].

**UCLA Loneliness Scale (UCLA-LS)**
Loneliness was assessed using the UCLA Loneliness Scale, consisting of 20 items rated on a 4-point scale (1–4). Higher scores denote stronger feelings of loneliness. The scale has shown excellent internal consistency (McDonald’s ωt=0.92)[11]. Its validity was generally acceptable, and exploratory analyses suggested a 2-factor structure accounting for 48.7% of the total variance[11].

**Interpersonal Trust Scale (ITS)**
Interpersonal trust was measured using the Interpersonal Trust Scale, which includes 25 items rated on a 5-point scale (1–5). Higher scores indicate greater levels of interpersonal trust. We used the Chinese translation compiled by Wang et al. (1999)[12]. A previous study using this Chinese version reported acceptable internal consistency (Cronbach α=0.70)[13].

**Social Avoidance and Distress Scale (SAD)**
Social avoidance and distress were assessed using the Social Avoidance and Distress Scale. The scale consists of 28 dichotomous items (True/False), with higher scores indicating more pronounced social avoidance and distress. The scale has shown good internal consistency (Cronbach α=0.85), and confirmatory factor analyses supported a 2-factor model in Chinese students[14].

**Description of Network and Longitudinal Modeling Procedures**

**CLPN Analysis**

Cross-Lagged Panel Networks (CLPNs) were estimated across three intervals (T0–T1, T1–T2, and T2–T3) using the Least Absolute Shrinkage and Selection Operator (LASSO) regression, in order to explore directional and potentially reciprocal associations among six prespecified variables: HAMA, HAMD-21, FFMQ, PSQI, S-AI, and PSS-10. These six variables were selected on theoretical and clinical grounds to balance conceptual relevance, statistical power, and model stability, thereby reducing the risk of overfitting given the available sample size.

To quantify how well each node was predicted by the others, node predictability (*R*²) was calculated. Network stability and accuracy were evaluated using 3,000 case-dropping bootstraps. Correlation stability (CS) coefficients were computed for both edge weights and node centrality indices. Edges that appeared in more than 90% of bootstrap resamples were interpreted as relatively stable and were used for descriptive interpretation. To aid interpretability and highlight directional dynamics, only cross-lagged paths with >90% bootstrap presence were visualized in the figures and discussed in the results. Autoregressive edges, although consistently strong, were omitted from figures due to their uniformly higher magnitude and limited theoretical interest in this context.

**RI-CLPM Analysis**

Random Intercept Cross-Lagged Panel Models (RI-CLPMs) were constructed to further examine selected directional pathways identified in the CLPN analyses within a framework that more explicitly accounts for stable between-person differences. This step was intended to evaluate whether selected associations remained consistent after more explicit modeling of stable trait-like variance, rather than to establish definitive causal relationships.

The RI-CLPM decomposed variance into a random intercept (representing between-person stability) and person-specific deviations (within-person fluctuations), thereby allowing a more explicit assessment of intraindividual temporal dynamics. To balance model complexity and estimation precision given the available sample size, we applied stationarity constraints by equating both autoregressive and cross-lagged parameters across time intervals. Although unstandardized coefficients (b) were constrained to be equal across waves, standardized coefficients (β) were allowed to vary due to differences in variable variance at each time point. Accordingly, mean β values were reported as summary measures across waves. We report both the common b estimate and the range of β values across intervals to facilitate interpretation.

Parameter estimation was conducted using robust maximum likelihood estimation to account for potential non-normality in the observed data. Model evaluation proceeded in three steps:

(a) Intraclass Correlation Coefficients (ICCs) were derived from the variance components to quantify the proportion of between-person stability relative to total variance.

(b) Model fit was assessed using commonly accepted benchmarks: Comparative Fit Index (CFI > 0.90), Root Mean Square Error of Approximation (RMSEA<0.08), and Standardized Root Mean Square Residual (SRMR<0.08).

(c) To contextualize the strength of within-person predictive paths, we referenced established effect size benchmarks for cross-lagged models, where standardized coefficients of 0.03, 0.07, and 0.12 were considered small, medium, and large, respectively.

**Supplementary Figures and Tables**

**Supplementary Figures**

**
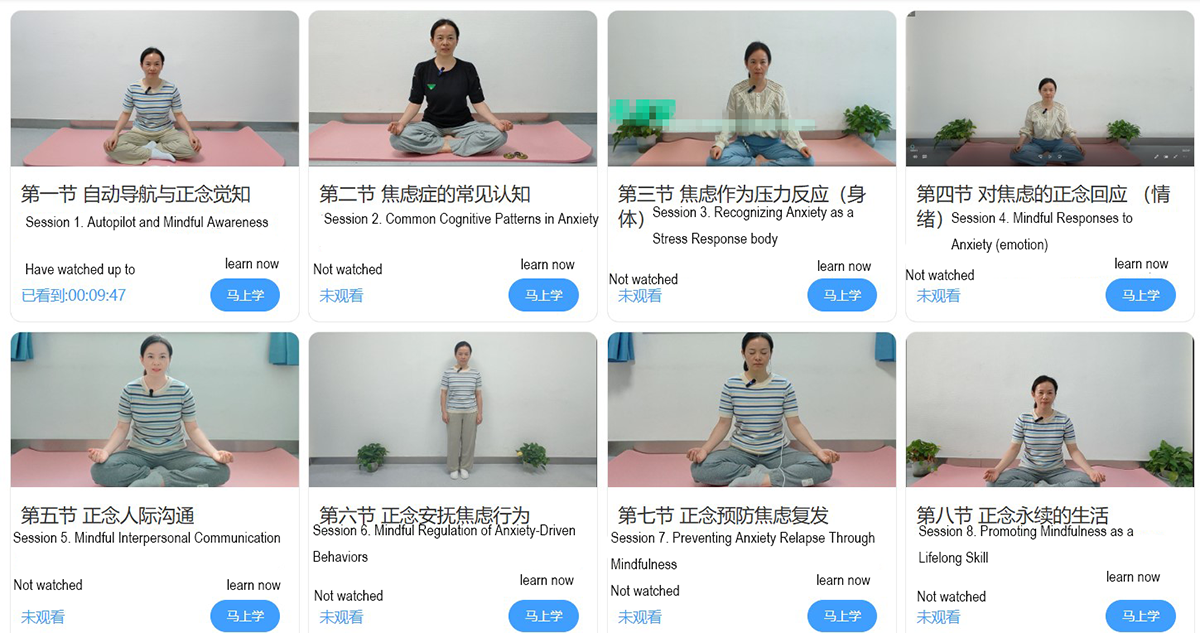
**

**Figure S1.** Overview of the 8-week internet-based mindfulness-informed stress management program used in this randomized controlled trial at Tongji Hospital.
The figure presents the structure and content of the eight-session web-based mindfulness program, including session themes and representative practice formats. The intervention sequentially targets attentional awareness, maladaptive cognitive patterns, stress-related bodily responses, emotional regulation, interpersonal mindfulness, behavioral regulation, relapse prevention, and the cultivation of mindfulness as a lifelong skill.


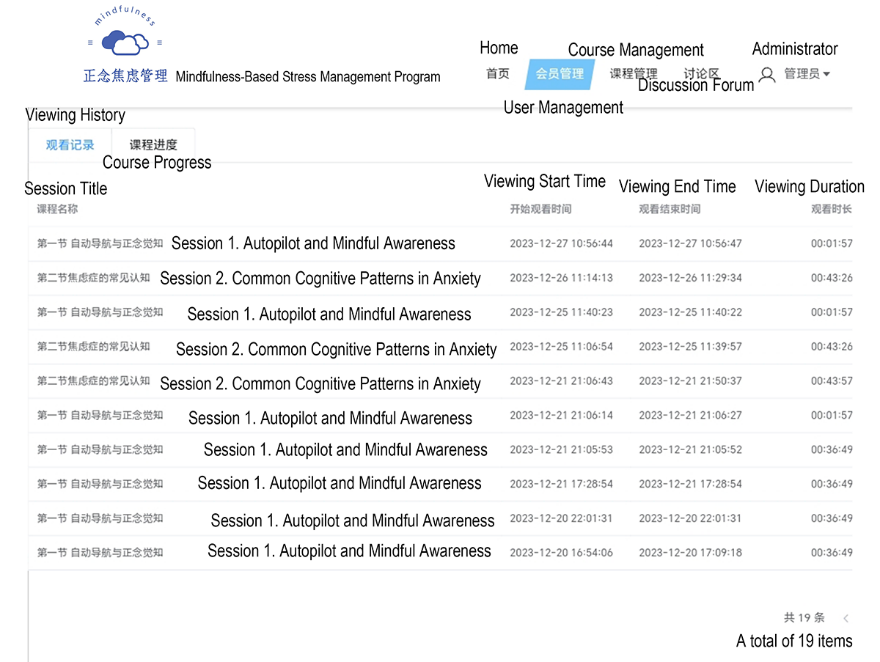


**Figure S2.** Screenshot of the course progress and viewing history interface of the internet-based mindfulness-informed stress management platform used in this randomized controlled trial at Tongji Hospital.
The interface displays participants’ session titles, viewing start and end times, and viewing duration, providing objective records of program engagement and adherence across intervention sessions.


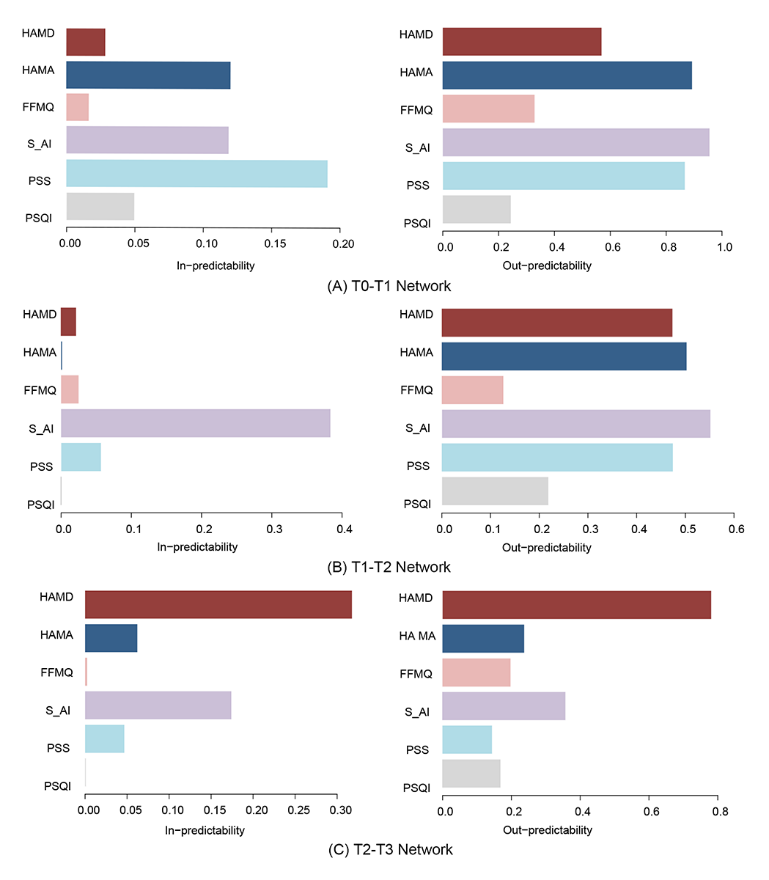


**Figure S3.** In-predictability and out-predictability of psychological variables across three consecutive temporal networks in participants with GAD in this randomized controlled trial at Tongji Hospital.
Panels display node-level in-predictability (left) and out-predictability (right) for each psychological variable in the (A) T0–T1, (B) T1–T2, and (C) T2–T3 networks. In-predictability reflects the proportion of variance in a given variable explained by other variables in the network at the previous time point, whereas out-predictability indicates the extent to which a variable predicts changes in other variables over time.
Abbreviations: GAD, generalized anxiety disorder; FFMQ, Five Facet Mindfulness Questionnaire; S-AI, State Anxiety Inventory; PSS, 10-item Perceived Stress Scale; HAMA, Hamilton Anxiety Rating Scale; HAMD, 21-item Hamilton Depression Rating Scale; PSQI, Pittsburgh Sleep Quality Index.

**Supplementary Tables**

**Table S1.** Overview of the internet-based mindfulness stress management program.

| **Week** | **Theme** | **Core Practices** |
| --- | --- | --- |
| 1 | Autopilot and Mindful Awareness | Intention exploration, raisin exercise, mindful stretching, loving-kindness meditation |
| 2 | Common Cognitive Patterns in Anxiety | Cognitive awareness practice, body scan, mindful stretching, loving-kindness meditation |
| 3 | Recognizing Anxiety as a Stress Response | Mindful breathing, body scan, mindful stretching, loving-kindness meditation |
| 4 | Mindful Responses to Anxiety | R.A.I.N. technique, mindful stretching, loving-kindness meditation |
| 5 | Mindful Interpersonal Communication | Mindful communication, mindful breathing, mindful stretching, loving-kindness meditation |
| 6 | Mindful Regulation of Anxiety-Driven Behaviors | Mindful breathing, mindful stretching, loving-kindness meditation |
| 7 | Preventing Anxiety Relapse Through Mindfulness | Mountain meditation |
| 8 | Promoting Mindfulness as a Lifelong Skill | Integrating mindfulness into daily life, writing a letter to oneself |

**Table S2.** Baseline demographic and clinical characteristics of participants with GAD in the iSM + TAU and TAU groups at Tongji Hospital (N=140).

| Variable | | All (N=140) | iSM + TAU (n=73) | TAU (n=67) | Test statistic | *P* value |
| --- | --- | --- | --- | --- | --- | --- |
| Age, years, median (IQR) | | 33 (26–39) | 33 (26–40) | 34 (26–39) | −0.084 | .93 |
| **Gender, n (%)** | |  |  |  | 0.031 | .86 |
|  | Male | 47 (33.6) | 25 (34.2) | 22 (32.8) |  |  |
|  | Female | 93 (66.4) | 48 (65.8) | 45 (67.2) |  |  |
| BMI, median (IQR) | | 21.28 (19.63–23.19) | 21.50 (19.52–23.15) | 21.09 (19.63–23.44) | −0.167 | .87 |
| **Education level, n (%)** | |  |  |  | 1.556 | .46 |
|  | Middle school or below | 16 (11.4) | 6 (8.2) | 10 (14.9) |  |  |
|  | High school | 15 (10.7) | 8 (11) | 7 (10.4) |  |  |
|  | College and above | 109 (77.9) | 59 (80.8) | 50 (74.6) |  |  |
| **Residential setting, n (%)** | |  |  |  | 2.472 | .30 |
|  | Urban | 114 (81.4) | 57 (78.1) | 57 (85.1) |  |  |
|  | Town | 19 (13.6) | 13 (17.8) | 6 (9) |  |  |
|  | Rural | 7 (5) | 3 (4.1) | 4 (6) |  |  |
| **Relationship status, n (%)** | |  |  |  | 2.179 | .55 |
|  | Married | 81 (57.9) | 40 (54.8) | 41 (61.2) |  |  |
|  | Partnered | 12 (8.6) | 5 (6.8) | 7 (10.4) |  |  |
|  | Single | 43 (30.7) | 25 (34.2) | 18 (26.9) |  |  |
|  | Divorced | 4 (2.9) | 3 (4.1) | 1 (1.5) |  |  |
| **Childhood parental separation, n (%)** | |  |  |  | 0.272 | .60 |
|  | Yes | 43 (30.7) | 21 (28.8) | 22 (32.8) |  |  |
|  | No | 97 (69.3) | 52 (71.2) | 45 (67.2) |  |  |
| **Employment status, n (%)** | |  |  |  | 0.052 | .97 |
|  | Student | 21 (15) | 11 (15.1) | 10 (14.9) |  |  |
|  | Employed | 95 (67.9) | 49 (67.1) | 46 (68.7) |  |  |
|  | Unemployed | 24 (17.1) | 13 (17.8) | 11 (16.4) |  |  |
| **Living arrangement, n (%)** | |  |  |  | 1.885 | .17 |
|  | Single-person household | 23 (16.4) | 15 (20.5) | 8 (11.9) |  |  |
|  | Shared household | 117 (83.6) | 58 (79.5) | 59 (88.1) |  |  |
| **Monthly household income, n (%)** | |  |  |  | 3.579 | .48 |
|  | <CNY 5000 (<US $732) | 9 (6.4) | 5 (6.8) | 4 (6) |  |  |
|  | CNY 5000–8300 (US $732–1215) | 21 (15) | 8 (11) | 13 (19.4) |  |  |
|  | CNY 8300–12,500 (US $1215–1830) | 40 (28.6) | 24 (32.9) | 16 (23.9) |  |  |
|  | CNY 12,500–24,000 (US $1830–3514) | 45 (32.1) | 25 (34.2) | 20 (29.9) |  |  |
|  | >CNY 24,000 (>US $3514) | 25 (17.9) | 11 (15.1) | 14 (20.9) |  |  |
| **Smoking or alcohol use history, n (%)** | |  |  |  | 0.001 | .97 |
|  | Yes | 27 (19.3) | 14 (19.2) | 13 (19.4) |  |  |
|  | No | 113 (80.7) | 59 (80.8) | 54 (80.6) |  |  |
| **Family history of psychiatric disorders, n (%)** | |  |  |  | 0.284 | .62 |
|  | Yes | 21 (15) | 12 (16.4) | 9 (13.4) |  |  |
|  | No | 119 (85) | 61 (83.6) | 58 (86.6) |  |  |
| **History of other chronic diseases, n (%)** | |  |  |  | 1.575 | .21 |
|  | Yes | 43 (30.7) | 19 (26) | 24 (35.8) |  |  |
|  | No | 97 (69.3) | 54 (74) | 43 (64.2) |  |  |
| **Medication status, n (%)** | |  |  |  | 0.254 | .61 |
|  | SSRIs only | 101 (72.1) | 54 (74) | 47 (70.1) |  |  |
|  | SSRIs + benzodiazepines | 39 (27.9) | 19 (26) | 20 (29.9) |  |  |
| **Baseline psychological measures** | |  |  |  |  |  |
|  | T-AI, mean (SD) | 50.17 (8.90) | 50.30 (8.72) | 50.03 (9.16) | −0.179 | .86 |
|  | PSSS, mean (SD) | 57.78 (12.63) | 57.85 (11.69) | 57.70 (13.67) | −0.068 | .95 |
|  | SQ, mean (SD) | 49.01 (11.74) | 48.88 (11.29) | 49.16 (12.29) | 0.144 | .89 |
|  | UCLA-LS, mean (SD) | 46.21 (10.13) | 46.56 (9.30) | 45.82 (11.02) | −0.428 | .67 |
|  | ITS, mean (SD) | 75.21 (7.96) | 74.36 (6.98) | 76.15 (8.86) | 1.336 | .18 |
|  | SAD, median (IQR) | 14.0 (8.0–22.0) | 14.0 (9.0–21.0) | 14.0 (7.5–23.0) | −0.142^a^ | .89 |

Abbreviations: GAD, generalized anxiety disorder; iSM, internet-based stress management program; TAU, treatment as usual; CNY, Chinese yuan; SSRIs, selective serotonin reuptake inhibitors; T-AI, Trait Anxiety Inventory; PSSS, Perceived Social Support Scale; SQ, Security Questionnaire; UCLA-LS, UCLA Loneliness Scale; ITS, Interpersonal Trust Scale; SAD, Social Avoidance and Distress Scale; IQR, interquartile range.

Continuous variables are presented as mean (SD) or median (IQR), as appropriate. Categorical variables are presented as n (%). Group comparisons for continuous variables were performed using independent-samples t tests or Mann–Whitney U tests, depending on data distribution. Categorical variables were compared using *χ²* tests.

ᵃ Mann–Whitney U test.

**Table S3.** Estimated group differences from linear mixed-effects models in the ITT analysis between the iSM + TAU and TAU groups among participants with GAD at Tongji Hospital (N=140).

| Outcome | | iSM + TAU Mean (SE) | TAU Mean (SE) | Interaction *P* Interaction Pᵃ | Mean difference (95% CI) | *P* valueᵇ | Cohen *d* (95% CI) |
| --- | --- | --- | --- | --- | --- | --- | --- |
| **Primary outcomes** | | |  |  |  |  |  |
| **HAMA** | |  |  |  |  |  |  |
|  | T0 | 18.37 (0.93) | 16.39 (0.78) | <.001 | 1.982 (−0.416 to 4.379) | .10 | 0.275 (−0.058 to 0.608) |
|  | T1 | 7.07 (0.48) | 9.54 (0.68) |  | −2.469 (−4.233 to −0.705) | .006 | −0.343 (−0.587 to −0.099) |
|  | T2 | 4.93 (0.41) | 6.93 (0.56) |  | −1.994 (−3.758 to −0.230) | .03 | −0.277 (−0.521 to −0.033) |
|  | T3 | 3.40 (0.35) | 7.07 (0.69) |  | −3.677 (−5.441 to −1.913) | <.001 | −0.511 (−0.755 to −0.267) |
| **HAMD**-**21** | |  |  |  |  |  |  |
|  | T0 | 16.66 (0.61) | 15.97 (0.66) | <.001 | 0.687 (−1.101 to 2.476) | .45 | 0.129 (−0.206 to 0.464) |
|  | T1 | 7.75 (0.46) | 9.79 (0.63) |  | −2.038 (−3.554 to −0.522) | .009 | −0.382 (−0.664 to −0.099) |
|  | T2 | 5.41 (0.38) | 7.06 (0.55) |  | −1.649 (−3.165 to −0.133) | .03 | −0.309 (−0.592 to −0.026) |
|  | T3 | 3.85 (0.34) | 7.25 (0.66) |  | −3.404 (−4.920 to −1.888) | <.001 | −0.637 (−0.920 to −0.355) |
| **Secondary outcomes** | | |  |  |  |  |  |
| **PHQ**-**15** | |  |  |  |  |  |  |
|  | T0 | 9.64 (0.51) | 9.13 (0.47) | <.001 | 0.510 (−0.851 to 1.870) | .46 | 0.125 (−0.209 to 0.459) |
|  | T1 | 4.33 (0.33) | 5.39 (0.40) |  | −1.059 (−2.136 to 0.017) | .05 | −0.260 (−0.523 to 0.003) |
|  | T2 | 2.84 (0.31) | 4.22 (0.38) |  | −1.388 (−2.465 to −0.312) | .01 | −0.340 (−0.604 to −0.077) |
|  | T3 | 2.30 (0.22) | 4.31 (0.43) |  | −2.012 (−3.088 to −0.936) | <.001 | −0.493 (−0.757 to −0.230) |
| **PSQI** | |  |  |  |  |  |  |
|  | T0 | 11.03 (0.45) | 11.03 (0.47) | <.001 | −0.002 (−1.279 to 1.275) | >.99 | −0.001 (−0.335 to 0.334) |
|  | T1 | 7.97 (0.43) | 8.81 (0.50) |  | −0.833 (−2.054 to 0.387) | .18 | −0.218 (−0.537 to 0.100) |
|  | T2 | 6.89 (0.36) | 7.15 (0.39) |  | −0.259 (−1.479 to 0.962) | .68 | −0.068 (−0.386 to 0.251) |
|  | T3 | 5.38 (0.36) | 7.63 (0.54) |  | −2.243 (−3.464 to −1.023) | <.001 | −0.588 (−0.906 to −0.269) |
| **SDSS** | |  |  |  |  |  |  |
|  | T0 | 4.10 (0.31) | 4.18 (0.38) | .13 | −0.083 (−1.055 to 0.888) | .87 | −0.029 (−0.364 to 0.307) |
|  | T1 | 1.68 (0.19) | 1.91 (0.24) |  | −0.226 (−0.893 to 0.442) | .51 | −0.078 (−0.308 to 0.152) |
|  | T2 | 1.23 (0.16) | 1.66 (0.21) |  | −0.424 (−1.091 to 0.243) | .21 | −0.146 (−0.376 to 0.083) |
|  | T3 | 0.90 (0.15) | 1.85 (0.22) |  | −0.947 (−1.614 to −0.279) | .006 | −0.327 (−0.557 to −0.097) |
| **S-AI** | |  |  |  |  |  |  |
|  | T0 | 47.42 (1.33) | 48.15 (1.46) | .04 | −0.725 (−4.629 to 3.180) | .71 | −0.062 (−0.397 to 0.273) |
|  | T1 | 41.75 (1.36) | 44.25 (1.40) |  | −2.500 (−6.152 to 1.151) | .18 | −0.215 (−0.527 to 0.098) |
|  | T2 | 37.16 (1.32) | 43.42 (1.22) |  | −6.254 (−9.905 to −2.602) | <.001 | −0.537 (−0.849 to −0.224) |
|  | T3 | 35.03 (1.23) | 39.64 (1.14) |  | −4.614 (−8.266 to −0.963) | .01 | −0.396 (−0.708 to −0.084) |
| **FFMQ** | |  |  |  |  |  |  |
|  | T0 | 116.66 (1.67) | 116.37 (1.32) | <.001 | 0.284 (−3.926 to 4.495) | .89 | 0.022 (−0.310 to 0.355) |
|  | T1 | 124.64 (1.53) | 120.22 (1.45) |  | 4.420 (0.106 to 8.734) | .045 | 0.349 (0.010 to 0.688) |
|  | T2 | 127.49 (1.54) | 119.06 (1.56) |  | 8.433 (4.119 to 12.748) | <.001 | 0.666 (0.327 to 1.006) |
|  | T3 | 131.15 (1.60) | 124.04 (1.65) |  | 7.106 (2.792 to 11.420) | .001 | 0.561 (0.222 to 0.901) |
| **RRS** | |  |  |  |  |  |  |
|  | T0 | 45.55 (1.42) | 49.25 (1.66) | .38 | −3.706 (−8.021 to 0.610) | .092 | −0.288 (−0.623 to 0.047) |
|  | T1 | 37.44 (1.19) | 39.57 (1.29) |  | −2.129 (−5.773 to 1.516) | .25 | −0.165 (−0.448 to 0.117) |
|  | T2 | 33.90 (1.24) | 38.33 (1.24) |  | −4.424 (−8.069 to −0.780) | .02 | −0.344 (−0.626 to −0.062) |
|  | T3 | 31.59 (1.10) | 36.60 (1.28) |  | −5.008 (−8.652 to −1.363) | .007 | −0.389 (−0.671 to −0.107) |
| **PSS**-**10** | |  |  |  |  |  |  |
|  | T0 | 21.25 (0.73) | 20.16 (0.83) | <.001 | 1.082 (−1.106 to 3.271) | .33 | 0.166 (−0.169 to 0.501) |
|  | T1 | 13.41 (0.70) | 15.96 (0.71) |  | −2.544 (−4.483 to −0.606) | .01 | −0.390 (−0.686 to −0.094) |
|  | T2 | 12.78 (0.70) | 15.58 (0.57) |  | −2.801 (−4.740 to −0.863) | .005 | −0.429 (−0.725 to −0.133) |
|  | T3 | 11.27 (0.70) | 14.34 (0.60) |  | −3.069 (−5.008 to −1.131) | .002 | −0.470 (−0.766 to −0.174) |

Abbreviations: ITT, intention-to-treat; iSM, internet-based mindfulness-informed stress management; TAU, treatment as usual; GAD, generalized anxiety disorder; HAMA, Hamilton Anxiety Rating Scale; HAMD-21, 21-item Hamilton Depression Rating Scale; PHQ-15, 15-item Patient Health Questionnaire; PSQI, Pittsburgh Sleep Quality Index; S-AI, State Anxiety Inventory;

FFMQ, Five Facet Mindfulness Questionnaire; PSS-10, 10-item Perceived Stress Scale; RRS, Ruminative Responses Scale; SDSS, Social Disability Screening Schedule.

Data are presented as estimated marginal means ± standard errors (SE) derived from linear mixed-effects models.

ᵃ Group × time interaction effects from linear mixed-effects models.

ᵇ Post hoc between-group comparisons (iSM + TAU minus TAU) with Bonferroni correction.

**Table S4.** Estimated group differences from linear mixed-effects models in the PP analysis between the iSM + TAU and TAU groups among participants with GAD at Tongji Hospital (n=95).

| Outcome | | iSM + TAU Mean (SE) | TAU Mean (SE) | Interaction *P* valueᵃ | Mean difference (95% CI) | *P* valueᵇ | Cohen *d* (95% CI) |
| --- | --- | --- | --- | --- | --- | --- | --- |
| **Primary outcomes** | | |  |  |  |  |  |
| **HAMA** | |  |  |  |  |  |  |
| T0 | | 17.52 (1.04) | 17.24 (1.02) | .08 | 0.275 (−2.620 to 3.169) | .85 | 0.039 (−0.369 to 0.446) |
| T1 | | 7.04 (0.60) | 8.80 (0.57) |  | −1.768 (−3.755 to 0.219) | .08 | −0.249 (−0.527 to 0.030) |
| T2 | | 4.57 (0.45) | 6.05 (0.60) |  | −1.475 (−3.461 to 0.512) | .15 | −0.207 (−0.486 to 0.071) |
| T3 | | 3.20 (0.38) | 5.98 (0.74) |  | −2.772 (−4.759 to −0.785) | .006 | −0.390 (−0.668 to −0.112) |
| **HAMD**–**21** | | |  |  |  |  |  |
|  | T0 | 16.17 (0.72) | 16.44 (0.80) | .07 | −0.272 (−2.404 to 1.859) | .80 | −0.053 (−0.463 to 0.358) |
|  | T1 | 7.65 (0.58) | 9.24 (0.60) |  | −1.596 (−3.336 to 0.144) | .07 | −0.308 (−0.641 to 0.026) |
|  | T2 | 5.09 (0.45) | 6.41 (0.70) |  | −1.322 (−3.062 to 0.418) | .14 | −0.255 (−0.589 to 0.079) |
|  | T3 | 3.67 (0.38) | 6.49 (0.74) |  | −2.821 (−4.561 to −1.081) | .002 | −0.544 (−0.878 to −0.210) |
| **Secondary outcomes** | | |  |  |  |  |  |
| **PHQ**–**15** | |  |  |  |  |  |  |
|  | T0 | 9.19 (0.53) | 9.44 (0.53) | .19 | −0.254 (−1.732 to 1.224) | .73 | −0.070 (−0.477 to 0.337) |
|  | T1 | 4.24 (0.38) | 4.88 (0.39) |  | −0.637 (−1.810 to 0.536) | .29 | −0.176 (−0.498 to 0.146) |
|  | T2 | 2.50 (0.29) | 3.80 (0.49) |  | −1.305 (−2.478 to −0.132) | .03 | −0.360 (−0.682 to −0.038) |
|  | T3 | 2.04 (0.24) | 3.61 (0.47) |  | −1.573 (−2.746 to −0.400) | .009 | −0.434 (−0.755 to −0.112) |
| **PSQI** | |  |  |  |  |  |  |
|  | T0 | 10.69 (0.57) | 11.10 (0.62) | .31 | −0.412 (−2.086 to 1.261) | .63 | −0.101 (−0.511 to 0.309) |
|  | T1 | 7.85 (0.52) | 8.63 (0.45) |  | −0.782 (−2.249 to 0.684) | .29 | −0.192 (−0.549 to 0.165) |
|  | T2 | 6.50 (0.43) | 7.10 (0.49) |  | −0.598 (−2.064 to 0.869) | .42 | −0.146 (−0.503 to 0.210) |
|  | T3 | 5.48 (0.42) | 7.05 (0.64) |  | −1.567 (−3.034 to −0.101) | .04 | −0.384 (−0.741 to −0.027) |
| **SDSS** | |  |  |  |  |  |  |
|  | T0 | 3.67 (0.36) | 4.02 (0.41) | .47 | −0.358 (−1.445 to 0.730) | .51 | −0.136 (−0.548 to 0.276) |
|  | T1 | 1.61 (0.22) | 1.49 (0.20) |  | 0.123 (−0.614 to 0.861) | .74 | 0.047 (−0.231 to 0.325) |
|  | T2 | 1.02 (0.18) | 1.29 (0.24) |  | −0.274 (−1.011 to 0.463) | .47 | −0.104 (−0.382 to 0.174) |
|  | T3 | 0.76 (0.17) | 1.32 (0.25) |  | −0.558 (−1.295 to 0.179) | .14 | −0.211 (−0.490 to 0.067) |
| **S-AI** | |  |  |  |  |  |  |
|  | T0 | 47.13 (1.54) | 47.85 (1.70) | .08 | −0.724 (−5.281 to 3.833) | .75 | −0.065 (−0.476 to 0.345) |
|  | T1 | 41.30 (1.53) | 45.54 (1.58) |  | −4.240 (−8.649 to 0.168) | .059 | −0.382 (−0.777 to 0.013) |
|  | T2 | 37.07 (1.52) | 43.32 (1.72) |  | −6.243 (−10.652 to −1.834) | .006 | −0.562 (−0.957 to −0.167) |
|  | T3 | 34.57 (1.42) | 40.17 (1.53) |  | −5.597 (−10.005 to −1.188) | .013 | −0.504 (−0.899 to −0.109) |
| **FFMQ** | |  |  |  |  |  |  |
|  | T0 | 119.43 (1.96) | 117.88 (1.73) | .009 | 1.548 (−3.640 to 6.736) | .56 | 0.121 (−0.284 to 0.525) |
|  | T1 | 125.59 (1.89) | 120.56 (2.04) |  | 5.032 (−0.559 to 10.623) | .08 | 0.392 (−0.040 to 0.824) |
|  | T2 | 128.85 (1.86) | 120.15 (2.18) |  | 8.706 (3.115 to 14.296) | .002 | 0.678 (0.246 to 1.110) |
|  | T3 | 132.26 (1.96) | 125.15 (2.18) |  | 7.113 (1.522 to 12.704) | .013 | 0.554 (0.122 to 0.986) |
| **RRS** | |  |  |  |  |  |  |
|  | T0 | 44.89 (1.71) | 48.88 (2.09) | .42 | −3.989 (−9.354 to 1.376) | .14 | −0.308 (−0.721 to 0.106) |
|  | T1 | 38.00 (1.45) | 39.71 (1.80) |  | −1.707 (−6.292 to 2.877) | .46 | −0.132 (−0.483 to 0.219) |
|  | T2 | 35.33 (1.54) | 36.95 (1.58) |  | −1.618 (−6.203 to 2.967) | .49 | −0.125 (−0.476 to 0.226) |
|  | T3 | 32.15 (1.34) | 36.37 (1.55) |  | −4.218 (−8.802 to 0.367) | .07 | −0.325 (−0.676 to 0.026) |
| **PSS**–**10** | |  |  |  |  |  |  |
|  | T0 | 20.56 (0.85) | 20.20 (0.91) | .03 | 0.360 (−2.108 to 2.829) | .77 | 0.060 (−0.350 to 0.470) |
|  | T1 | 14.00 (0.81) | 16.12 (0.87) |  | −2.122 (−4.527 to 0.283) | .08 | −0.352 (−0.749 to 0.045) |
|  | T2 | 12.81 (0.90) | 15.41 (0.73) |  | −2.600 (−5.005 to −0.195) | .03 | −0.432 (−0.829 to −0.035) |
|  | T3 | 11.24 (0.85) | 14.05 (0.83) |  | −2.808 (−5.213 to −0.403) | .02 | −0.466 (−0.863 to −0.069) |

Abbreviations: PP, per-protocol; iSM, internet-based stress management program; TAU, treatment as usual; GAD, generalized anxiety disorder; HAMA, Hamilton Anxiety Rating Scale; HAMD-21, 21-item Hamilton Depression Rating Scale; PHQ-15, 15-item Patient Health Questionnaire; PSQI, Pittsburgh Sleep Quality Index; S-AI, State Anxiety Inventory; FFMQ, Five Facet Mindfulness Questionnaire; PSS-10, 10-item Perceived Stress Scale; RRS, Ruminative Responses Scale; SDSS, Social Disability Screening Schedule.

Data are presented as estimated marginal means (SE) derived from linear mixed-effects models.

ᵃ *P* value for the group × time interaction term.

ᵇ Bonferroni-adjusted *P* value for post hoc between-group comparisons.

**Table S5.** Distribution of completed intervention sessions in the iSM + TAU group (n=73)

| **Sessions completed** | **Participants, n (%)** |
| --- | --- |
| <1 completed session | 7 (9.6) |
| 1 | 5 (6.8) |
| 2 | 4 (5.5) |
| 3 | 3 (4.1) |
| 4 | 8 (11) |
| 5 | 5 (6.8) |
| 6 | 4 (5.5) |
| 7 | 11 (15.1) |
| 8 | 26 (35.6) |

**Supplementary Results**

**Variance Decomposition and Autoregressive Effects**

**Model 1: Perceived Stress (PSS) and State Anxiety (S-AI)**

**Model Fit Indices**

The model demonstrated excellent fit to the data (*χ²*_13_=19.1; CFI=0.983; TLI=0.963; RMSEA=0.058, SRMR=0.047). All fit indices met or exceeded recommended thresholds, indicating excellent correspondence between the model and observed data.

**Variance Decomposition (Intraclass Correlation Coefficients)**

ICC values indicated that approximately 51.7% of the variance in PSS and 49.1% of the variance in S-AI was attributable to stable between-person differences, with the remaining variance reflecting within-person fluctuations across time.

**Between-Person Associations**

At the between-person level, the random intercepts of PSS and S-AI were strongly positively correlated (r=0.654, *P*<.001), indicating that individuals with chronically higher perceived stress consistently reported higher state anxiety throughout the study period.

**Within-Person Autoregressive Effects**

No significant autoregressive effects were observed for PSS (b=0.018, SE=0.059, 95% CI −0.098 to 0.133, *P*=.77) or S-AI (b=−0.129, SE=0.073, 95% CI −0.272 to 0.013, *P*=.08), suggesting that within-person deviations in these constructs showed limited temporal stability after accounting for between-person differences (Table 3).

**Model 2: Mindfulness and State Anxiety**

**Model Fit Indices**

The model demonstrated excellent fit to the data (*χ²*_13_=23.6, CFI=0.983; TLI=0.963; RMSEA=0.076; SRMR=0.075). All fit indices met or exceeded recommended thresholds, indicating excellent correspondence between the model and observed data.

**Variance Decomposition (Intraclass Correlation Coefficients)**

ICC values revealed that 68.5% of the variance in FFMQ and 48.3% of the variance in S-AI was attributable to stable between-person differences, with the remaining variance reflecting within-person fluctuations across time. The substantially higher ICC for mindfulness suggests it represents a more stable individual characteristic compared to state anxiety.

**Between-Person Associations**

At the between-person level, the random intercepts of FFMQ and S-AI were moderately negatively correlated (r*=*−0.505, *P*<.001), indicating that individuals with chronically higher mindfulness consistently reported lower state anxiety throughout the study period.

**Within-Person Autoregressive Effects**

For FFMQ, significant autoregressive effects emerged (b=0.362, SE=0.086, 95% CI 0.193 to 0.531, *P*<.001), indicating strong within-person temporal stability in mindfulness. In contrast, autoregressive effects for S-AI were not statistically significant (b=−0.124, SE=0.073, 95% CI −0.266 to 0.019, *P*=.09), though the negative trend approached significance (Table 3).

References

[1] Zhang, M.; He, Y. Handbook of Rating Scales in Psychiatry. *Hunan Science and Technology Press, Changsha*, **1998**.

[2] Zheng, Y. P.; Zhao, J. P.; Phillips, M.; Liu, J. B.; Cai, M. F.; Sun, S. Q.; Huang, M. F. Validity and Reliability of the Chinese Hamilton Depression Rating Scale. *Br J Psychiatry*, **1988**, *152*, 660–664. https://doi.org/10.1192/bjp.152.5.660.

[3] Ma, W.-F.; Liu, Y.-C.; Chen, Y.-F.; Lane, H.-Y.; Lai, T.-J.; Huang, L.-C. Evaluation of Psychometric Properties of the Chinese Mandarin Version State-Trait Anxiety Inventory Y Form in Taiwanese Outpatients with Anxiety Disorders. *J Psychiatr Ment Health Nurs*, **2013**, *20* (6), 499–507. https://doi.org/10.1111/j.1365-2850.2012.01945.x.

[4] Deng, Y.-Q.; Liu, X.-H.; Rodriguez, M. A.; Xia, C.-Y. The Five Facet Mindfulness Questionnaire: Psychometric Properties of the Chinese Version. *Mindfulness*, **2011**, *2* (2), 123–128. https://doi.org/10.1007/s12671-011-0050-9.

[5] Han, X.; Yang, H. Chinese Version of Nolen-Hoeksema Ruminative Responses Scale (RRS) Used in 912 College Students: Reliability and Validity. *Chinese Journal of Clinical Psychology*, **2009**, *17* (5), 550–551.

[6] Lu, W.; Bian, Q.; Wang, W.; Wu, X.; Wang, Z.; Zhao, M. Chinese Version of the Perceived Stress Scale-10: A Psychometric Study in Chinese University Students. *PLoS One*, **2017**, *12* (12), e0189543. https://doi.org/10.1371/journal.pone.0189543.

[7] Zhang, L.; Fritzsche, K.; Liu, Y.; Wang, J.; Huang, M.; Wang, Y.; Chen, L.; Luo, S.; Yu, J.; Dong, Z.; et al. Validation of the Chinese Version of the PHQ-15 in a Tertiary Hospital. *BMC Psychiatry*, **2016**, *16*, 89. https://doi.org/10.1186/s12888-016-0798-5.

[8] Lu, T.; Li, Y.; Xia, P.; Zhang, G.; Wu, D. Analysis on Reliability and Validity of the Pittsburgh Sleep Quality Index. *Chongqing medicine*, **2014**, 260–263.

[9] Zhong, C.; Lijuan, A. Developing of Security Questionnaire and Its Reliability and Validity. *Chinese Mental Health Journal*, **2004**.

[10] Fan, Z.; Shuzhen, Z.; Ping, D. Evaluation of Perceived Social Support Scale Used in Study of Social Support among Hospitalized Patients in China. *Chinese Nursing Research*, **2018**, *32* (13).

[11] Ip, H.; Suen, Y. N.; Hui, L. M. C.; Cheung, C.; Wong, S. M. Y.; Chen, E. Y. H. Psychometric Properties of the Variants of the Chinese UCLA Loneliness Scales and Their Associations with Mental Health in Adolescents. *Sci Rep*, **2024**, *14*, 24663. https://doi.org/10.1038/s41598-024-75739-w.

[12] Xiangdong, W.; Xilin, W.; Hong, M. Manual of Mental Health Rating Scale. *Chinese Journal of Mental Health*, **1999**, No. Suppl, 31–35.

[13] Tang, M.; Lu, T.; You, X. From Initial to Situational Automation Trust: The Interplay of Personality, Interpersonal Trust, and Trust Calibration in Young Males. *Behav Sci (Basel)*, **2026**, *16* (2), 176. https://doi.org/10.3390/bs16020176.

[14] Chun - zi, P.; Xiao - ling, F.; Luo - chu, L. The Validity and Reliability of Social Avoidance and Distress Scale in Chinese Students. *Chinese Journal of Clinical Psychology*, **2003**, *11* (4), 279–281.
